# Hydrogen-bond bridging dually facilitates exciton dissociation and charge migration for enhanced photocatalytic water oxidation

**DOI:** 10.1093/nsr/nwaf392

**Published:** 2025-09-17

**Authors:** Jianfang Jing, Xinyue Tan, Jingyi Xu, Wenting Li, Yiguo Su, Yongfa Zhu

**Affiliations:** College of Chemistry and Chemical Engineering, Inner Mongolia University, Hohhot 010021, China; College of Chemistry and Chemical Engineering, Inner Mongolia University, Hohhot 010021, China; Department of Chemistry, Tsinghua University, Beijing 100084, China; Department of Chemistry, Tsinghua University, Beijing 100084, China; College of Chemistry and Chemical Engineering, Inner Mongolia University, Hohhot 010021, China; Department of Chemistry, Tsinghua University, Beijing 100084, China

**Keywords:** photocatalysis, oxygen evolution, hydrogen bond, exciton dissociation, perylene diimide

## Abstract

As a kinetic bottleneck in artificial photosynthesis, the hole-mediated oxygen evolution reaction (OER) remains constrained by inefficient charge separation. We demonstrate here a hydrogen-bonded perylene diimide/aminated-fullerene (PDINH/C_60_NH_3_) supramolecular photocatalyst that concurrently enhances exciton dissociation efficiency (from 39.5% to 70.5%) and charge migration. The hydrogen bond between fullerene-tethered protonated amino and PDINH carbonyls creates a polarized bridge that enables exciton delocalization and conversion into weakly bound charge-transfer excitons, resulting in a significant decrease of exciton binding energy from 76.5 meV to 50.9 meV. Also, hydrogen-bond–induced charge polarization enhances the internal electric field by 3.5-fold, thereby accelerating charge migration and yielding a 19-fold increase in surface-reaching holes. Strikingly, PDINH/C_60_NH_3_ achieves a state-of-the-art OER rate of 63.9 mmol g^−1^ h^−1^ with remarkable apparent quantum efficiencies (11.83% at 420 nm and 4.08% at 650 nm). This hydrogen-bond–bridged charge separation establishes a new paradigm for enhancing solar-to-chemical conversion efficiency.

## Teaser

Hydrogen-bonded polarized bridge constructed between supramolecular perylene diimide and protonated aminated-fullerene synergistically reduces exciton binding energy while enhancing charge transfer efficiency, achieving exceptional photocatalytic oxygen evolution performance.

## INTRODUCTION

The oxygen evolution reaction (OER) is crucial in clean energy storage and conversion, as it facilitates the generation of protons and electrons from water. Particularly in photocatalytic overall water splitting, the hole-mediated OER serves as an indispensable half reaction for maintaining charge balance and mitigating carrier recombination through continuous hole consumption, thereby ensuring efficient and sustained water splitting [1–3]. Nevertheless, supplying four holes for the oxidation reaction is thermodynamically more challenging than two electrons for the reduction reaction. Furthermore, water oxidation involves a four-electron-transfer process with multiple intermediate transformations, resulting in inherently sluggish kinetics compared to proton reduction, which significantly restricts the overall water splitting efficiency [4–6]. Therefore, the development of high-performance photocatalysts for oxygen evolution and mechanism elucidation of hole-mediated water oxidation become imperative for advancing water-splitting technologies.

Recently, perylene diimide-based systems have emerged as promising photocatalysts for water oxidation, owing to their broad spectral absorption, sufficiently deep valence band and excellent stability [7–9]. In particular, supramolecular perylene diimide (PDINH) with high crystallinity synthesized by the imidazole solvent method has demonstrated attractive oxygen evolution performance [10]. However, the homogeneous charge distribution in PDINH results in an intrinsically weak dipole moment and negligible polarization effects, resulting in inefficient photoexcited charge separation and migration, which fundamentally limits further enhancement of its water oxidation activity. Moreover, organic semiconductors typically exhibit large exciton binding energies that significantly impedes electron-hole pair dissociation and limits the generation of free charge carriers [11,12], leading to suboptimal photocatalytic performance. Besides, illumination at the photocatalyst–solution junction causes a thermal non-equilibrium between hole and electron concentrations due to phonon-assisted relaxation of photogenerated charge carriers [13]. Under this imbalance, excessive electron accumulation suppresses hole survival and interfacial extraction, ultimately reducing the available hole concentration at the surface and thus impeding water oxidation kinetics [14]. Therefore, improving exciton dissociation efficiency and enhancing hole availability in PDINH remain critical yet challenging issues, with the underlying mechanisms still not fully elucidated.

Although the rational substituent modification in supramolecular monomer has been well explained in promoting charge separation [15,16], introducing functional groups into PDINH monomer will disrupt the high crystallinity of their aggregates, consequently impairing charge transport. To address the above problems in the PDINH system, we propose a post-assembly modification strategy that exploits the abundant O atoms in PDINH as reactive sites. Specifically, amino-functionalized fullerenes are anchored onto the PDINH through directed hydrogen bonding (N–H–H···O) between amino and carbonyl. This hydrogen-bonded PDINH/fullerene composite offers two key advantages. First, the excellent electron extraction/transporting ability of fullerenes enables their widespread application as electron acceptors, effectively promoting charge separation [17,18]. Second, the protonated amino-functionalized fullerenes act as hydrogen bond donors, establishing hydrogen-bonding bridges that would promote exciton delocalization and modulate exciton binding energies [19,20]. In addition, the hydrogen bond could enhance electrostatic potential and induce charge polarization, thereby strengthening the internal electric field that accelerates the migration of charge carriers [21].

In view of the above, we functionalized fullerene through ethylenediamine modification followed by acidification (referred to herein as C_60_NH_3_) to provide protonated amino groups for hydrogen bonding with the carbonyl of PDINH. *In situ* experimental characterizations combined with density functional theory (DFT) calculations verify the formation of strong N–H–H···O hydrogen bonds. Furthermore, this hydrogen-bond bridging facilitates the conversion of tightly bound excitons into weakly bound charge-transfer excitons, thereby reducing the exciton binding energy and consequently improving exciton dissociation efficiency. Meanwhile, hydrogen-bond–induced strong polarization enhances the internal electric field that accelerates directional charge migration. Benefitting from the synergistic enhancement of exciton dissociation and charge migration, the hydrogen-bonded PDINH/C_60_NH_3_ achieves a remarkable oxygen evolution rate of 63.9 mmol g^−1^ h^−1^, with an apparent quantum efficiency (AQE) of 11.83% at 420 nm. Notably, the system maintains a high AQE of 4.08% even at 650 nm, ranking it among the top-performing organic photocatalysts for water oxidation across the broad visible spectrum.

## RESULTS AND DISCUSSION

### Formation of hydrogen bond in PDINH/C_60_NH_3_

**Figure 1.**
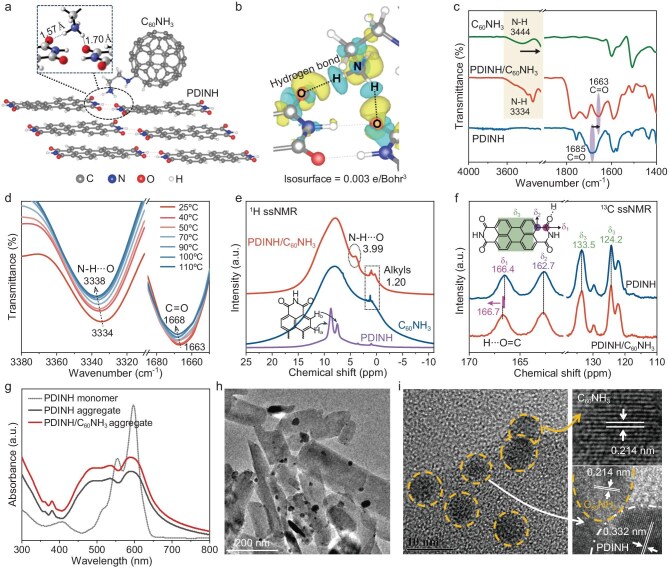
Structural characterizations of hydrogen-bonded PDINH/C_60_NH_3_. (a) Geometric structure of PDINH/C_60_NH_3_ and schematic illustration of hydrogen bond. (b) Differential charge density map of PDINH/C_60_NH_3_. The blue and yellow areas represent electronic deficiency and accumulation, respectively. (c) FT-IR spectra. (d) Temperature-dependent FT-IR spectra of PDINH/C_60_NH_3_. (e) Solid-state ^1^H NMR spectra. (f) Solid-state ^13^C NMR spectra. (g) UV-vis absorption spectra. (h) TEM image of PDINH/C_60_NH_3_. (i) HRTEM image (right side shows local magnification) of PDINH/C_60_NH_3_.

The PDINH was prepared through the imidazole solvent method (Fig. S1) [10]. To precisely anchor fullerenes onto the oxygen sites of PDINH, ethylenediamine was bonded to fullerenes via an addition reaction followed by protonation in hydrochloric acid, yielding the functionalized fullerenes, defined as C_60_NH_3_ (Fig. S2). This modification enables the formation of an intermolecular hydrogen bond (N–H···O) between the protonated amino groups and PDINH carbonyl moieties, thereby constructing the PDINH/C_60_NH_3_ composite [22]. The Zeta potentials reveal a negatively charged surface of PDINH and a positively charged surface of C_60_NH_3_ (Fig. S3), and this electrostatic complementarity promotes spontaneous attraction and interfacial contact between PDINH and C_60_NH_3_ [23]. DFT calculations were employed to demonstrate the formation of hydrogen bonds (Figs S4 and S5). As observed in Fig. 1a, the H atoms in the –NH_3_ group of C_60_NH_3_ form dual interactions with adjacent PDINH molecules through N–H–H···O bonds of 1.70 Å and 1.57 Å. Furthermore, the differential charge density maps intuitively demonstrate the pronounced charge polarization of N–H in C_60_NH_3_ and C=O in PDINH, showing electron deficiency of H atoms and electron accumulation at O atoms (Fig. 1b and Fig. S6), confirming the strong N–H–H···O hydrogen bonding. To gain insight into the hydrogen bonding mechanism, we employed ethylenediamine-modified fullerenes without protonation (C_60_NH_2_) and non-functionalized fullerenes (C_60_) as two comparative models. Structural optimization reveals that PDINH/C_60_NH_2_ forms a weaker N–H–H···N hydrogen bond of 1.84 Å between the H atom in the imide group (–CONH) of PDINH and N atom of C_60_NH_2_ (Figs S7 and S8). Obviously, there are no hydrogen atoms available in pristine C_60_ to form hydrogen bonds with PDINH. Besides, the PDINH/C_60_NH_3_ configuration exhibits minimum energy compared with the other two counterparts, suggesting that N–H–H···O hydrogen bonding contributes to the thermodynamic stability (Fig. S7) [24].

The hydrogen bond is further confirmed by Fourier transform infrared (FT-IR) spectra. As shown in Fig. 1c, upon integration with PDINH, the characteristic N–H stretching vibration in C_60_NH_3_ (∼3444 cm^−1^) exhibits a significant red-shift of ∼100 cm^−1^ with broadening and intensified absorption. Simultaneously, the C=O stretching vibration in PDINH also undergoes a red-shift of ∼20 cm^−1^, definitively evidencing the formation of a N–H–H···O hydrogen bond between –NH_3_ and C=O [25,26]. Also, X-ray photoelectron spectroscopy (XPS) of O 1s demonstrates a higher binding energy for the hydrogen-bonded carbonyl in PDINH/C_60_NH_3_ (Fig. S9), which is consistent with the observed IR spectral shifts. In addition, the diffraction peaks show a slight low-angle shift upon C_60_NH_3_ incorporation, suggesting a perturbation in the molecular stacking configuration induced by hydrogen bonding interactions (Fig. S10). Notably, temperature-dependent IR spectra further corroborate hydrogen bonding in PDINH/C_60_NH_3_. Both N–H and C=O vibrations exhibit blue-shift and weakened intensity with increasing temperature, evidently confirming the occurrence of N–H–H···O hydrogen bonds (Fig. 1d, Figs S11 and S12). This thermal response behavior is consistent with the gradual breakage of intermolecular hydrogen bonds at elevated temperatures [27]. Moreover, solid-state nuclear magnetic resonance (ssNMR) measurements were performed to examine the N–H–H···O bond. As shown in Fig. 1e, the ^1^H ssNMR spectrum of PDINH contains two typical peaks at 8.77 ppm and 7.52 ppm, corresponding to the H_a_ of bay-position and H_b_ of α-position, respectively. The broad peak at ∼8.01 ppm in C_60_NH_3_ can be assigned to the fullerene-bound protons, while the overlapping high-field peaks at ∼1.20 ppm correspond to alkyl chains and amino protons [28]. Significantly, a new downfield-shifted signal at 3.99 ppm appears in PDINH/C_60_NH_3_ but is absent in PDINH/C_60_NH_2_ (Fig. S13), indicating N–H–H···O hydrogen bond formation. This conclusion is further supported by a downfield shift of carbonyl carbon resonance (δ_1_) in the ^13^C ssNMR spectrum (Fig. 1f). This phenomenon arises from the enhanced electron-withdrawing effect of the hydrogen-bonded carbonyl oxygen, which increases electron deshielding at the carbonyl carbon nucleus [29]. The resonances at δ_2_ and δ_3_ are assigned to the α-carbon adjacent to the carbonyl group and the perylene diimide conjugated backbone, respectively, and these characteristic peaks show no significant shifts. Element analysis demonstrates a significantly higher N/C weight ratio in PDINH/C_60_NH_3_ (0.134) compared to pristine PDINH (0.088) (Table S1), indicating the successful immobilization of amino-functionalized fullerenes onto the supramolecular PDINH. This finding further aligns with the relative N/C ratio obtained from XPS analysis (Table S2).

In addition, the hydrogen bonding in PDINH/C_60_NH_3_ is further reflected by absorption spectra. Upon incorporation of C_60_NH_3_, the composite exhibits a blue-shift and intensified absorption in the visible range, coincident with the absorption spectral characteristics of the hydrogen-bonded supramolecular materials (Fig. 1g and Fig. S14) [30]. The morphology and microstructure of PDINH/C_60_NH_3_ were investigated by transmission electron microscopy (TEM) and high-resolution TEM (HRTEM). As presented in Fig. 1h, Figs S15 and S16, C_60_NH_3_ nanoparticles (∼22 nm average size) are uniformly dispersed on the PDINH surface and in intimate contact, while no clear close contact is observed between the two components in either PDINH/C_60_ or PDINH/C_60_NH_2_ (Fig. S17). HRTEM images reveal distinct lattice fringes of 0.332 nm and 0.214 nm (Fig. 1i), corresponding to the π-π stacking distance of PDINH and the characteristic d-spacing of C_60_NH_3_, respectively. This observation confirms the successful immobilization of C_60_NH_3_ on the PDINH surface. Combining the above analyses, the hydrogen bond formation between the fullerene-bonded amino group and carbonyl oxygen of PDINH is unambiguously identified.

### Hydrogen-bond–facilitated exciton delocalization and charge-transfer exciton formation

**Figure 2.**
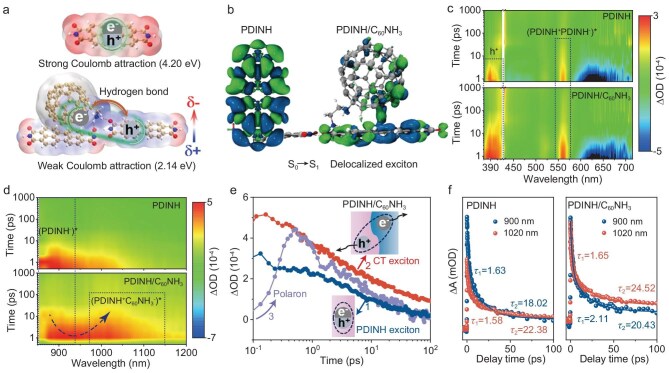
DFT calculation and fs-TA analysis for exciton delocalization. (a) Electrostatic potential distribution and calculated Coulomb attraction energy between electron-hole pairs. (b) S_0_→S_1_ excited state of PDINH and PDINH/C_60_NH_3_. The blue and green isosurface represents holes and electrons, respectively. (c) Femtosecond transient absorption in visible light region. (d) Femtosecond transient absorption in near-infrared range. (e) Time evolution of exciton absorption and polaron generation in PDINH/C_60_NH_3_. (f) TA decay dynamics of PDINH and PDINH/C_60_NH_3_ at 900 nm and 1020 nm.

We then sought to elucidate the role of the hydrogen bond in exciton dissociation within the PDINH/C_60_NH_3_ system. Molecular electrostatic potential calculations reveal that the hydrogen-bonded PDINH/C_60_NH_3_ generates a pronounced charge-polarized environment. This enhanced polarization strengthens dielectric screening [19,31], thereby reducing the Coulombic attraction energy between electron-hole pairs from 4.20 eV to 2.14 eV (Fig. 2a). In contrast, PDINH/C_60_ lacking hydrogen bonds shows weak polarization and consequently maintains a much higher Coulomb attraction energy of 3.60 eV (Fig. S18). Furthermore, the strong polarization effect promotes exciton delocalization via hydrogen-bond bridging, converting tightly bound Frenkel excitons into weakly bound charge-transfer (CT) excitons [32]. Time-dependent DFT calculations of the S_0_→S_1_ excitation reveal significant exciton delocalization in PDINH/C_60_NH_3_, in contrast to the localized excitons confined in pristine PDINH (Fig. 2b and Fig. S19).

More specifically, femtosecond transient absorption spectroscopy (fs-TA) offers deep insight into the exciton dissociation dynamics within PDINH/C_60_NH_3_, revealing the critical timescales and pathways for charge separation. As shown in Fig. 2c, the TA spectrum displays two distinct excited-state absorption (ESA) features in the visible region, a band at 370–410 nm and a feature at ∼560 nm originating from the polarized exciton state (PDINH^+^PDINH^−^)^∗^ formed through S_1_→S_N_ transitions between PDINH moieties [33,34]. When ascorbic acid (AA) is introduced as a hole scavenger, the 370–410 nm signal shows significant quenching, whereas electron scavenger (AgNO_3_) produces negligible effects. This selective quenching unambiguously identifies the 370–410 nm absorption as surface-trapped holes (Fig. S20). Notably, PDINH/C_60_NH_3_ exhibits enhanced and broadened ESA signals relative to pristine PDINH, reflecting extended exciton lifetime and more efficient charge photogeneration. The negative signal observed in the 600–700 nm region overlaps with both the UV-vis absorption and photoluminescence (PL) spectrum, indicating its combined contributions from ground-state bleach (GSB) and stimulated emission (SE) (Fig. S21). Global analysis of the TA data in the 570–780 nm region reveals significantly varying kinetic processes, where the fast-decaying component aligns with SE, while the slowest decay component (>1 ns) corresponds to long-lived GSB (Fig. S22). This prolonged GSB decay indicates the formation of CT excitons at the PDINH/C_60_NH_3_ interface, which effectively delays charge recombination to the ground state [35,36].

Furthermore, near-infrared TA spectra of PDINH/C_60_NH_3_ reveals a characteristic absorption at 1020 nm, attributed to CT excitons (PDINH^+^C_60_NH_3_^−^)^∗^ [37,38], which develops concomitantly with attenuation and red-shift of the ESA signal at ∼900 nm (Fig. 2d). In contrast, the pristine C_60_NH_3_ exhibits a singlet excited state absorption maximum at ∼1150 nm (Fig. S23). Compared to this reference, the 1020 nm ESA in PDINH/C_60_NH_3_ shows a significant blue-shift, further providing evidence for CT exciton formation across the donor-acceptor interface. However, PDINH/C_60_ without hydrogen bonding shows negligible ESA features in this spectral region (Fig. S24), demonstrating that hydrogen-bond–mediated exciton delocalization is crucial for facilitating CT exciton formation.

Kinetic analysis of exciton absorption reveals the exciton conversion and dissociation in hydrogen-bonded PDINH/C_60_NH_3_. As shown in Fig. 2e, the tightly bound excitons originating from the PDINH aggregate excitation (blue line) delocalize via hydrogen-bond–bridging, converting into CT excitons (red line). The CT excitons then spontaneously dissociate into charge carriers, manifested as a rising polaron absorption within ∼0.5 ps (purple line) (Fig. S25). Furthermore, the extracted TA decay curves at 900 nm and 1020 nm were compared between pristine PDINH and PDINH/C_60_NH_3_ (Fig. 2f). Obviously, the TA lifetime of PDINH/C_60_NH_3_ is longer than that of the PDINH due to the formation of CT excitons. Notably, PDINH/C_60_NH_3_ exhibits an ∼0.5 ps shorter lifetime (*τ*_1_) at 1020 nm than that at 900 nm, suggesting faster dissociation of CT excitons into free carriers compared to localized exciton states in pristine PDINH. Meanwhile, the longer lifetime of *τ*_2_ indicates extended diffusion and survival of photogenerated electrons in the hydrogen-bonded PDINH/C_60_NH_3_ system.

### Lowered exciton binding energy enables efficient spontaneous exciton dissociation

**Figure 3.**
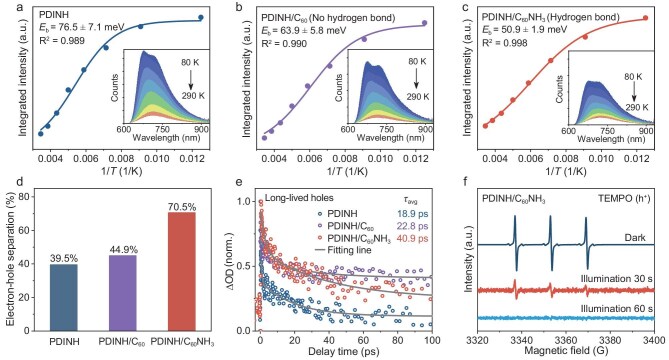
Enhanced electron-hole separation efficiency in hydrogen-bonded PDINH/C_60_NH_3_. (a–c) Fitted exciton binding energy from the corresponding temperature-dependent PL spectra (inset) of (a) PDINH, (b) PDINH/C_60_ and (c) PDINH/C_60_NH_3_. (d) Separation efficiency of electron-hole pairs. (e) Fitting results of hole decay dynamics. (f) *In-situ* EPR spectra of photogenerated holes scavenging TEMPO radicals.

Furthermore, temperature-dependent PL spectroscopy was performed to investigate the effect of exciton delocalization on electron-hole separation dynamics. As displayed in Fig. 3a–c, the integrated PL intensity decreases with increasing temperature from 80 K to 290 K. The exciton binding energy (*E*_b_) was evaluated by fitting these data using the Arrhenius equation (Fig. S26) [39]. Notably, the hydrogen-bonded PDINH/C_60_NH_3_ possesses a substantially lower *E*_b_ (50.9 meV) compared to both pristine PDINH (76.5 meV) and the non-hydrogen-bonded PDINH/C_60_ (63.9 meV). This suggests that hydrogen-bond–bridged exciton delocalization plays a decisive role in reducing the binding energy. The low exciton binding energy facilitates spontaneous exciton dissociation, generating more free charge carriers available for photocatalytic reactions. Accordingly, we conducted a comparative analysis of the electron-hole separation efficiencies across the three systems using the established method of Li and co-workers, wherein both electron acceptors (Fe^3+^) and hole acceptors (CH_3_OH) were added simultaneously in a photocatalytic water splitting reaction (Figs S27 and S28) [40]. As shown in Fig. 3d, the charge separation efficiency of the PDINH/C_60_NH_3_ system was estimated to be 70.5%, significantly surpassing pristine PDINH (39.5%) and non-hydrogen-bonded PDINH/C_60_ (44.9%). This efficiency trend directly correlates with exciton binding energies, confirming that hydrogen-bond–mediated low exciton binding energy effectively improves dissociation efficiency. Furthermore, Fig. 3e compares the hole decay kinetics by fitting the normalized TA profiles probed at 398 nm with a bi-exponential function. The average lifetime (*τ*_avg_) of holes for PDINH/C_60_NH_3_ (40.9 ps) is remarkably longer than those of PDINH (18.9 ps) and PDINH/C_60_ (22.8 ps), indicating that hydrogen bonding facilitates efficient exciton-to-carrier dissociation. To directly probe the carrier concentrations, we performed *in situ* electron paramagnetic resonance (EPR) measurements under light irradiation, employing 2,2,6,6-tetramethylpiperidine-1-oxyl (TEMPO) as a hole-trapping agent (Fig. 3f and Fig. S29) [41]. TEMPO itself has a characteristic triplet EPR signal under dark, whereas upon illumination, the EPR signal intensity decreases due to oxidation of TEMPO by photoinduced holes. Impressively, the EPR signal intensity of PDINH/C_60_NH_3_ shows rapid quenching, decreasing substantially within 30 s and completely vanishing by 60 s of illumination, while PDINH and PDINH/C_60_ exhibit significantly slower decay under identical conditions. The rapid quenching of the EPR signal further confirms enhanced generation of free charge carriers in PDINH/C_60_NH_3_, resulting from its efficient exciton dissociation.

### Hydrogen-bond–enhanced internal electric field drives efficient charge migration

**Figure 4.**
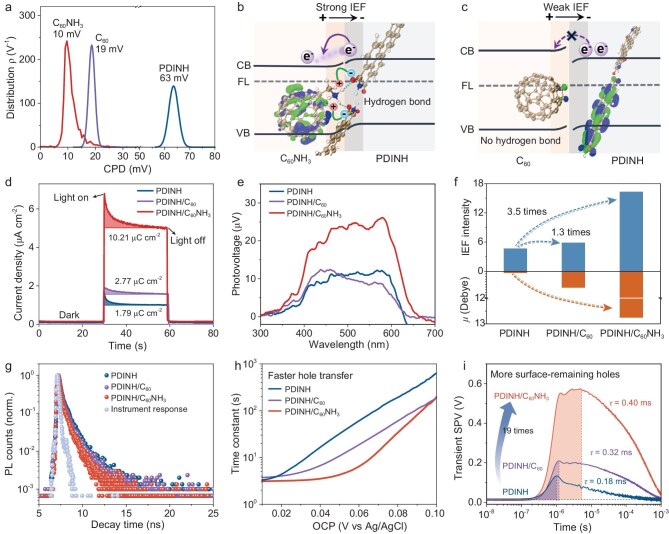
Enhanced internal electric field and accelerated charge migration. (a) Contact potential differences between samples and Pt probe by KPFM measurement. (b) Interfacial photogenerated charge migration of PDINH/C_60_NH_3_. FL, Fermi level; CB, conduction band minimum; VB, valence band maximum. (c) Interfacial photogenerated charge migration of PDINH/C_60_. (d) Surface charge density measured by transient photocurrent. (e) SPV spectra. (f) Comparison of internal electric field intensity. (g) Time-resolved PL decay spectra. (h) Time constants of hole extraction estimated from open-circuit photovoltage decay. (i) Transient photovoltage spectra with 355 nm laser excitation.

Given the demonstrated efficient electron-hole separation and increased dissociated-charges resulting from lowered exciton binding energy in PDINH/C_60_NH_3_, the charge migration dynamics was further explored. To delve into the role of hydrogen bonding in charge migration, the work functions (*Φ*), directly related to the Fermi level (FL), were determined by measuring the contact potential differences (CPDs) between samples and probe using *in situ* Kelvin probe force microscopy (KPFM) [42,43]. As shown in Fig. 4a, both C_60_NH_3_ (10 mV) and C_60_ (19 mV) present significantly lower CPD than PDINH (63 mV), corresponding to work functions of 3.88 eV, 3.89 eV and 3.93 eV, respectively (see details in Figs S30 and S31). Combined with diffuse reflectance spectroscopy (DRS) and Mott–Schottky measurements, the energy band structures and FL alignments are depicted in Figs S32 and S33. The higher Fermi levels of C_60_NH_3_ (3.88 eV) and C_60_ (3.89 eV) relative to PDINH (3.93 eV) drive spontaneous electron transfer from C_60_NH_3_/C_60_ to PDINH upon contact, until their Fermi levels reach thermodynamic equilibrium [44]. Consequently, the interfacial energy bands bend accordingly, establishing an internal electric field (IEF) directed from C_60_NH_3_/C_60_ to PDINH (Fig. 4b, c and Fig. S34), which drives the directional migration of photogenerated electrons and holes. Notably, PDINH/C_60_NH_3_ contact exhibits the minimal energy-level offset (0.15 eV) compared to PDINH/C_60_ (0.34 eV) (Fig. S33). This appropriate band arrangement favors efficient electron transfer from PDINH to C_60_NH_3_ with minimum energy loss. Furthermore, the hydrogen bond induces strong charge polarization in PDINH/C_60_NH_3_, enabling a markedly larger dipole moment (μ  =  12.8 D) compared to both pristine PDINH and PDINH/C_60_ (bottom in Fig. 4f). This enhanced dipole moment strengthens the IEF in PDINH/C_60_NH_3_. Additionally, frontier molecular orbital analysis reveals distinct spatial separation in PDINH/C_60_NH_3_, where the lowest unoccupied molecular orbital is localized on C_60_NH_3_ while the highest occupied molecular orbital resides on PDINH. In contrast, both orbitals remain predominantly on the PDINH moiety in PDINH/C_60_ (Fig. S35). This pronounced donor-acceptor interaction in PDINH/C_60_NH_3_ facilitates more efficient charge transfer. We then quantitatively evaluated the IEF intensity through combined surface charge density and surface photovoltage (SPV) measurements [22,45]. Transient photocurrent integration reveals a significantly higher surface charge density for PDINH/C_60_NH_3_ than that for PDINH/C_60_ and PDINH (Fig. 4d). SPV spectra demonstrate PDINH/C_60_NH_3_ generates an ∼2.5-fold stronger surface voltage relative to both control systems (Fig. 4e). Consequently, quantitative comparison of IEF magnitudes reveals that hydrogen-bonded PDINH/C_60_NH_3_ achieves a 3.5-fold enhancement compared to pristine PDINH, whereas the non-hydrogen-bonded PDINH/C_60_ shows only a 1.3-fold improvement (Fig. 4f). The enhanced IEF was further corroborated by surface potential (Δ*E*) measurements under dark conditions using KPFM. PDINH/C_60_NH_3_ exhibits a significantly higher Δ*E* (49 mV) compared to PDINH (18 mV) and PDINH/C_60_ (26 mV) (Fig. S36), confirming the hydrogen-bonding–enhanced IEF.

Furthermore, time-resolved photoluminescence (TR-PL) spectra were employed to investigate the charge migration dynamics in PDINH/C_60_NH_3_. As shown in Fig. 4g, the TR-PL decay profiles follow biexponential kinetics, characterized by a fast component (*τ*_1_, interfacial charge transport) and a slow component (*τ*_2_, nonradiative recombination) (Table S3). Notably, PDINH/C_60_NH_3_ displays a shortened average PL lifetime (0.386 ns) compared to PDINH (0.548 ns) and PDINH/C_60_ (0.482 ns), indicating accelerated electron transfer [46,47] driven by strong IEF. Also, the reduced charge transfer resistance (*R*_SC_), enhanced photocurrent response and pronounced PL quenching of PDINH/C_60_NH_3_ collectively reflect its efficient charge migration efficiency (Fig. S37 and Table S4). Consequently, efficient exciton dissociation and charge migration synergistically promote both hole transfer kinetics and the available hole concentration, thereby facilitating a water oxidation reaction. The hole extraction time constant (*τ*_h_), which inherently correlated with an actual photocatalytic reaction, was estimated through open-circuit photovoltage (OCP) decay measurements [14]. Notably, PDINH/C_60_NH_3_ exhibits a shorter *τ*_h_ relative to PDINH and PDINH/C_60_ (Fig. 4h and Fig. S38), suggesting enhanced interfacial hole transfer efficiency and consequent surface hole accumulation. We employed transient photovoltage (TPV) spectroscopy to further investigate charge transfer dynamics. As shown in Fig. 4i, PDINH/C_60_NH_3_ exhibits ∼47-fold and ∼24-fold enhancements in hole extraction amounts (*A*, shaded area) compared to pristine PDINH and PDINH/C_60_, respectively. Concurrently, the carrier recombination in PDINH/C_60_NH_3_ is significantly suppressed, as evidenced by an extended TPV decay time (*τ*  =  0.40 ms). Consequently, the effective surface hole concentration (*A*_e_) in PDINH/C_60_NH_3_ after charge extraction and recombination is determined to be 19-fold and 6-fold enhancements over PDINH and PDINH/C_60_, respectively [41] (Fig. S39 and Table S5). Together, these results demonstrate that the hydrogen bond in PDINH/C_60_NH_3_ promotes charge migration through a strong internal electric field, thus significantly enhancing the surface hole concentration available for the water oxidation reaction.

### Synergistic exciton dissociation and charge migration boost photocatalytic oxygen evolution

**Figure 5.**
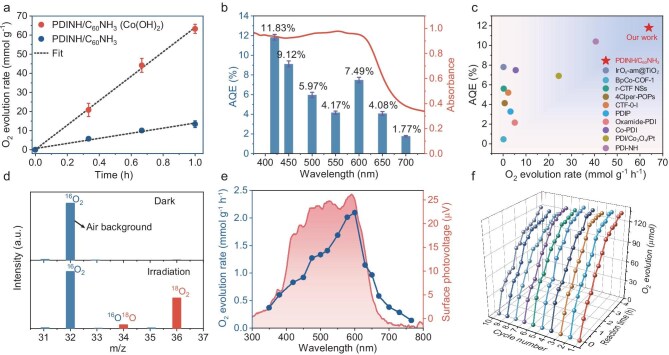
Improved photocatalytic O_2_ evolution performance of hydrogen-bonded PDINH/C_60_NH_3_. (a) Photocatalytic O_2_ evolution rate of PDINH/C_60_NH_3_ under visible light. (b) The AQE and absorption spectrum of PDINH/C_60_NH_3_. (c) Comparison of AQE at 420 nm and O_2_ evolution rate for the reported photocatalysts (summarized in detail in Table S7). (d) The mass spectrum of oxygen released during photocatalytic H_2_^18^O splitting. (e) Correlation between SPV signal and wavelength-dependent O_2_ evolution. (f) Recycling experiments of O_2_ evolution reaction for PDINH/C_60_NH_3_.

Bearing the efficient exciton dissociation and improved hole availability in mind, we systematically evaluated the photocatalytic O_2_ evolution performance for PDINH/C_60_NH_3_ under visible light irradiation (478 mW/cm^2^). Since the OER is a kinetically sluggish four-electron transfer process, it typically requires co-catalysts to reduce activation barriers and accelerate reaction kinetics. Layered Co(OH)_2_ has been widely employed as an effective OER co-catalyst, where its abundant surface hydroxyl groups promote reactant adsorption, while the facile redox transitions between multiple Co oxidation states provide highly active centers [48,49]. These combined advantages significantly enhance OER kinetics, justifying the selection of Co(OH)_2_ as a co-catalyst in this photocatalytic system. Under optimal conditions with 3 wt% Co(OH)_2_ and 20 mM AgNO_3_ as electron sacrificial agent, PDINH/C_60_NH_3_ achieves an exceptional O_2_ evolution rate of 63.9 mmol g^−1^ h^−1^ (Fig. 5a and Figs S40–S44). It should be noted that even without a co-catalyst, the O_2_ evolution of PDINH/C_60_NH_3_ (13.3 mmol g^−1^ h^−1^) is still ∼2 times higher than that of pristine PDINH (Fig. S45). This performance trend is in line with the co-catalyst-assisted systems, indicating that the introduction of C_60_NH_3_ dominates the performance enhancement independent of the presence of the co-catalyst. Furthermore, compared with two counterparts of PDINH/C_60_ and PDINH/C_60_NH_2_, PDINH/C_60_NH_3_ exhibits remarkably enhanced photocatalytic O_2_ evolution performance, proving that the water oxidation enhancement depends critically on N–H–H···O hydrogen bond formation (Fig. S46). Remarkably, PDINH/C_60_NH_3_ achieves an apparent quantum efficiency (AQE) of 11.83% at 420 nm, maintaining 4.08% even at 650 nm, which represents a 4.9-fold enhancement compared to pristine PDINH at this longer wavelength (Fig. 5b, Fig. S47 and Table S6). This superior O_2_ evolution performance of PDINH/C_60_NH_3_ is among the highest reported for organic semiconductors in water oxidation (Fig. 5c and Table S7). During the photocatalytic H_2_^18^O splitting process over PDINH/C_60_NH_3_, the labelled ^18^O_2_ with an m/z of 36 is clearly detected, whereas no ^18^O_2_ signal is observed under dark conditions. The observed ordinary ^16^O_2_ signal comes from unavoidable environmental background (Fig. 5d). This isotopic measurement unambiguously confirms that the evolved O_2_ is produced from light-driven water splitting. PDINH/C_60_NH_3_ yields strong positive SPV signals at 300–750 nm, suggesting enhanced hole accumulation on the surface (Fig. 4e). The wavelength-dependent O_2_ evolution activity closely correlates with SPV response, confirming that photogenerated holes drive the efficient water oxidation reaction (Fig. 5e). In addition, a photocatalytic stability test for PDINH/C_60_NH_3_ was performed. After 10 individual cycles for 40-h photocatalysis, the O_2_ evolution rate of PDINH/C_60_NH_3_ still retains ∼93% of the original value (Fig. 5f). However, during prolonged continuous operation, the O_2_ evolution rate gradually decreases and eventually reaches a plateau (Fig. S48), which is attributed to the progressive blockage of active sites through excessive Ag deposition (from Ag^+^ photoreduction). Post-reaction structural characterizations demonstrate the formation and distribution of abundant Ag nanoparticles on the catalyst surface (Figs S49–S51). Notably, the oxygen evolution activity is completely restored after Ag removal by nitric acid treatment (Fig. S48). Owing to its outstanding water oxidation activity, broad spectral absorption, and robust photostability, PDINH/C_60_NH_3_ holds great potential for coupling with hydrogen-producing catalysts to drive efficient overall water splitting.

## CONCLUSION

In summary, the integration of C_60_NH_3_ with supramolecular PDINH achieves a photocatalytic oxygen evolution rate of 63.9 mmol g^−1^ h^−1^, with AQE of 11.83% at 420 nm and 4.08% at 650 nm, representing one of the most efficient organic photocatalysts for water oxidation. The exceptional performance originates from the strong N–H–H···O hydrogen bonding that creates a charge-transfer bridge for exciton delocalization, thereby reducing exciton binding energy while enhancing electron-hole separation. Concurrently, the hydrogen bond induces a strengthened internal electric field, promoting directional charge carrier migration. These synergistic effects facilitate exciton-to-carrier conversion and increase hole availability, thus dramatically improving oxygen evolution activity. The hydrogen-bond–mediated exciton dissociation mechanism demonstrated here establishes a meaningful design paradigm for highly efficient photocatalytic water oxidation systems.

## Supplementary Material

nwaf392_Supplemental_File
