# Supplementary material for: Hydrogen-bond bridging dually facilitates exciton dissociation and charge migration for enhanced photocatalytic water oxidation
Source: Natl Sci Rev. 2025 Sep 17;12(12):nwaf392. doi: 10.1093/nsr/nwaf392 (PMC12669891; doi:10.1093/nsr/nwaf392)
Supplement: nwaf392_Supplemental_File [file nwaf392_supplemental_file.pdf]

Supplementary data

Hydrogen-bond bridging dual-facilitates exciton dissociation and charge migration for enhanced photocatalytic oxygen evolution

Jianfang Jing[1†*], Xinyue Tan[1†], Jingyi Xu[2], Wenting Li[2], Yiguo Su[1*], Yongfa Zhu[2*]

[1]College of Chemistry and Chemical Engineering, Inner Mongolia University, Hohhot 010021, P.R. China.

[2]Department of Chemistry, Tsinghua University, Beijing 100084, P.R. China. ***Corresponding authors.** Email: jingjf@imu.edu.cn (J. Jing); cesyg@imu.edu.cn (Y. Su); zhuyf@tsinghua.edu.cn (Y. Zhu).

[†]These authors contributed equally to this work.

Table of Contents

Experimental section 4

1.1 Materials 4

1.2 Synthesis of PDINH: 4

1.3 Synthesis of $C_{60}NH_3$: 4

1.4 Synthesis of PDINH/$C_{60}NH_3$: 5

1.5 Characterizations and Instrumentations 5

1.6 Transient photovoltage (TPV) measurements 6

1.7 Femtosecond transient absorption spectra (fs-TA) 6

1.8 Photoelectrochemical measurements 6

1.9 Photocatalytic oxygen evolution experiments 7

1.10 Computational methods 7

Supplemental Figures 8

Figure S1. Structural characterizations of PDINH. 8

Figure S2. Structural characterizations of $C_{60}NH_3$ 9

Figure S3. Zeta potentials 9

Figure S4-S7. DFT calculation analysis 10

Figure S8. Structural analysis of PDINH/$C_{60}NH_2$. 11

Figure S9. XPS analysis of PDINH/$C_{60}NH_3$. 12

Figure S10. XRD analysis PDNH/$C_{60}NH_3$ 13

Figure S11-S12. Temperature-dependent FT-IR spectra 13

Figure S13. Solid-state ^{1}H NMR spectra. 14

Figure S14. UV-Vis absorption spectra. 14

Figure S15-S17. Morphological characterizations 15

Figure S18. DFT calculated molecular electrostatic potentials distribution. 16

Figure S19. TD-DFT calculated excited states 17

Figure S20-S25. Femtosecond transient absorption analysis 17

Figure S26. Temperature-dependent PL spectra 20
Figure S27-S28. Quantitative analysis of Fe^{2+} 21
Figure S29. EPR detection........ 22
Figure S30. Contact potential difference measured by KPFM........ 23
Figure S31. The DFT calculated work functions........ 24
Figure S32-S34. Energy level alignment........ 25
Figure S35. DFT calculated frontier molecular orbitals........ 27
Figure S36. Surface potentials by KPFM........ 28
Figure S37. Characterizations of charge transfer. 28
Figure S38. The open-circuit photovoltage measurement........ 29
Figure S39. TPV dynamic analysis 29
Figure S40. Photocatalytic reaction equipment and O_2 calibration curve........ 30
Figure S41-S43. Optimization of oxygen evolution performance 30
Figure S44. Oxygen evolution detected by gas chromatography........ 32
Figure S45. The time-dependent O_2 evolution 32
Figure S46. OER performance comparison of PDINH-based composites........ 33
Figure S47. AQE of photocatalytic O_2 evolution for PDINH. 33
Figure S48. Long-term photocatalytic reaction test. 33
Figure S49-S51. Structural characterizations of post-reaction catalyst........ 34

Supplemental Tables 35

Table S1. Elemental analysis........ 35
Table S2. XPS analysis results of C/N/O elements. 35
Table S3. The TR-PL spectra data........ 35
Table S4. The fitted parameters of electrochemical impedance spectra. 35
Table S5. Two-exponential decay time constant of transient photovoltage........ 36
Table S6. The AQE of PDINH/$C_{60}NH_3$. 36
Table S7. Summary and comparison of photocatalytic oxygen-producing materials. 37

Supplemental References 38

Experimental section

1.1 Materials

All chemicals used in this work are analytical pure without further purification. The 3,4,9,10-perylenetetracarboxylic dianhydride (PTCDA) was purchased from J&K Scientific. The imidazole and ammonium sulfate [$(NH_4)_2SO_4$] were supplied by Shanghai Macklin Biochemical Technology Co., Ltd. The fullerenes (C_{60}) were obtained from Innochem. The ethylenediamine ($C_2H_8N_2$), cobalt nitrate [$Co(NO_3)_2$] and silver nitrate ($AgNO_3$) were acquired from Tianjin Fengchuan Chemical Reagent Co., Ltd. The lanthanum oxide (La_2O_3) was purchased from Aladdin.

1.2 Synthesis of PDINH:

The supramolecular PDINH was synthetized referring to the previous report (Scheme S1) [1]. $(NH_4)_2SO_4$ (16 mmol, 2.114 g) and PTCDA (2.0 mmol, 0.78 g) were dispersed in imidazole (25.0 g). The mixture was stirred at 150 ℃ for 4 hours, cooled to room temperature, and then treated with 4 M HCl (100 mL) under stirring for 5 hours. The dark red product was collected by centrifugation, washed with deionized water until the supernatant reached pH 7, and dried overnight at 60 ℃.

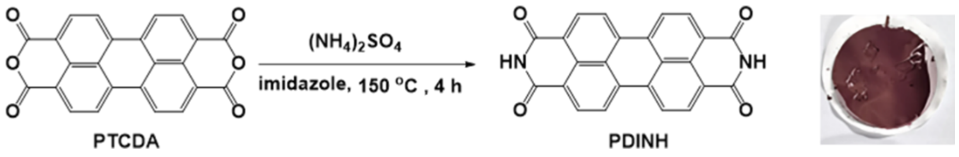

Scheme S1. The synthesis route of supramolecular PDINH.

1.3 Synthesis of $C_{60}NH_3$:

The protonated ethylenediamine-functionalized fullerenes ($C_{60}NH_3$) was synthesized according to the previous report with slight modifications (Scheme S2) [2]. 20 mg of fullerene and 100 mL of ethylenediamine were mixed in a round-bottomed flask and stirred at 50 ℃ for 24 hours. The mixture was then sonicated for 5 hours until complete dissolution. Ethylenediamine was removed by rotary evaporation, and the residual solid was dissolved in 100 mL of 1 M HCl to yield a $C_{60}NH_3$ solution with a concentration of 0.2 mg mL^{-1}. The synthesis of ethylenediamine-functionalized fullerenes ($C_{60}NH_2$) followed the same procedure as for $C_{60}NH_3$, except the residual solid was dissolved in 100 mL of deionized water instead of HCl solution.

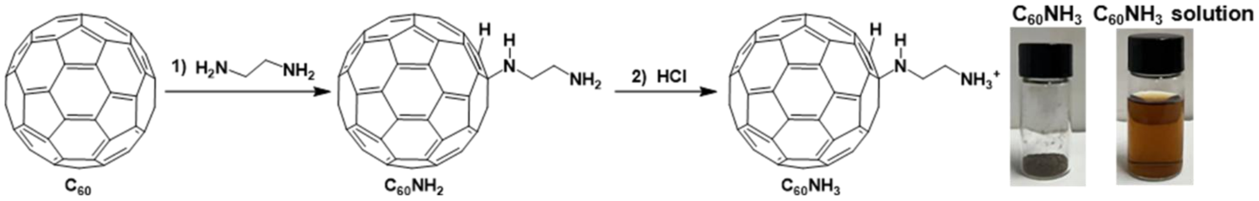

Scheme S2. Solid-liquid reaction to prepare $C_{60}NH_3$.

1.4 Synthesis of PDINH/$C_{60}NH_3$:

To prepare the composites, varying volumes (3, 3.5, 4, 4.5, and 5 mL) of the 0.2 mg mL^{-1} $C_{60}NH_3$ stock solution were added to 60 mg of PDINH (Scheme S3). The mixture was then dispersed in 20 mL of deionized water, sonicated for 30 minutes, and stirred for an additional 30 minutes. The final product was thoroughly washed with deionized water, then collected by filtration and dried at 60 ℃. The synthesis of PDINH/$C_{60}NH_2$ follows the same procedure as that of PDINH/$C_{60}NH_3$.

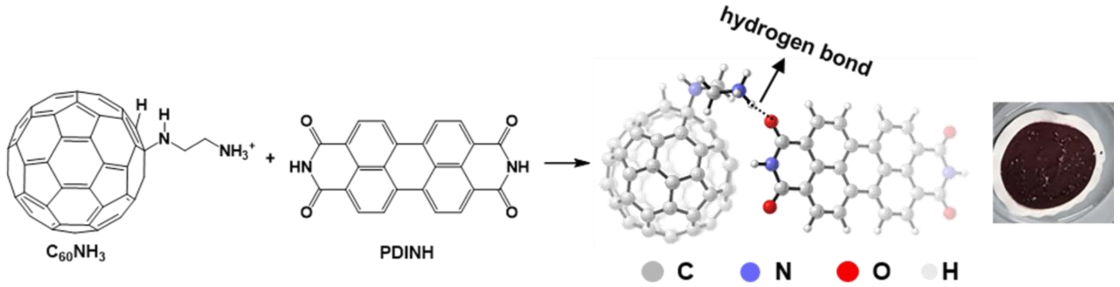

Scheme S3. The hydrogen bonding self-assembly between PDINH and $C_{60}NH_3$.

1.5 Characterizations and Instrumentations

The powder X-ray diffraction (XRD) patterns were obtained on PANalytical B.V. Empyrean at 40 kV and 40 mA with Cu Kα1 radiation. X-ray photoelectron spectroscopy (XPS) measurements were performed using an ESCALAB Xi+ instrument (Thermo Scientific) with the excitation source of monochromatized Al Kα. The morphology was observed by field emission transmission electron microscopy (TEM, FEI Tecnai F20, 200 kV). The high-resolution transmission electron microscopy (HRTEM) images were obtained by JEOL JEM-2100F. The Fourier transform infrared (FT-IR) spectra were acquired on Thermo Scientific Nicolet iN10 spectrometer. The optical properties were characterized by UV-Vis diffuse reflectance spectroscopy (DRS) on PerkinElmer LAMBDA 750 spectrophotometer with $BaSO_4$ as a reference. The atomic force microscopy with Kelvin probe (HQ NSC18/Pt) (KPFM, Oxford Cypher VRS) was used to measure the surface potentials of samples in the light and dark. The EPR and in-situ EPR tests were measured by a Bruker EMX plus EPR spectrometer (9.4 GHz). The solid-state nuclear magnetic resonance (ssNMR) spectroscopy was conducted on 400 MHz WB solid-state nuclear magnetic resonance spectrometer (Bruker AVANCE NEO 400 WB, CP MAS TOSS-4mm-8KHz-AVANCE NEO probe). The photoluminescence (PL) spectra were measured

by lifetime and steady state fluorescence spectrometer (Edinburgh FLS980) with an excitation wavelength of 403 nm. The surface photovoltage (SPV) measurements were conducted with a home-built instrument. Monochromatic light resource was a 500 W xenon lamp produced by Beijing Changtuo Company. The photovoltage signal was amplified by a lock-in amplifier (SR830-DSP) with a light chopper (SR540). The resolution of spectrum was 1 nm. The raw surface photovoltage data were normalized using the illuminometer (Zolix UOM-1S).

1.6 Transient photovoltage (TPV) measurements

TPV measurements were employed to investigate the kinetic of photogenerated charge separation, diffusion and recombination using CEL-TPV1000. The samples were excited with a laser radiation pulse at 355 nm and pulse width of 1.5 ns from a third-harmonic Nd:YAG laser (Polaris II, New Wave Research, Inc.). The TPV signal was recorded with a 200 MHz digital phosphor oscilloscope (PicoScope 5244D).

1.7 Femtosecond transient absorption spectra (fs-TA)

The laser source of the homemade spectroscopy setup is a commercial femtosecond amplifier laser system (35 fs, 1 kHz, 800 nm, Spitfire Ace, Spectra Physics). The output pulse was split into two beams. The first beam was used to pump a commercial wavelength conversion system (TOPAS prime and wavelength mixing unit from Spectra Physics) which will output tunable femtosecond laser pulses from 350 nm to 2600 nm. The pulses centered at 420 nm were selected as the excitation pulse. The second beam with weaker energy was focused on a CaF_2 plate (4 mm thickness) or an yttrium aluminium garnet plate to generate a white light continuum for the visible or near-infrared probe, respectively. The time delay between the pump beam and the probe pulses was controlled by a motorized delay stage. A laser frequency synchronized fibre optical spectrometer (AvaSpec-ULS2048CL-EVO, Avantes) was used to collect the probe light in range of 350-850 nm. A homemade 46-channel lock-in amplifier coupled spectrometer was utilized to collect the near-infrared transient signals (900-1350 nm). During the experiment, the pump intensity was set to be 340 μJ for the 420 nm laser pulses [3]. The samples were sonicated in deionized water (1×10^{-5} M) for fs-TA testing.

1.8 Photoelectrochemical measurements

Photoelectrochemical measurements were performed on a Swiss Aptar electrochemical workstation using a standard three-electrode cell with a working electrode (ITO glass), a platinum wire counter electrode and an Ag/AgCl reference electrode. A 300 W Xe lamp with cut-off filter ($\lambda \geq 420$ nm) was used as the light source and 0.2 M Na_2SO_4 aqueous solution was used as electrolyte solution. The sample powder (2 mg) was dispersed in H_2O (5 mL) to obtain a slurry, and then the slurry was coated onto of the prerinsed ITO glass (1×1 cm^2) and dried in an oven at 80 ℃ for 2 h. Electrochemical impedance spectroscopy (EIS) spectra were recorded under an AC perturbation signal of 10 mV over

the frequency range from 100 kHz to 0.1 Hz. Mott-Schottky plots were collected with a scan rate of 5 mV s^{-1} at 800, 1000 and 1500 Hz.

1.9 Photocatalytic oxygen evolution experiments

The photocatalytic oxygen evolution reaction was performed in a Pyrex top-irradiation reaction vessel with a stationary temperature at 5°C, which was connected to a glass closed gas system (Labsolar-6A, Beijing Perfectlight). All the reaction solutions were kept at 5°C by recirculating cooling water system, then it was evacuated several times to remove air completely. The amounts of gases produced were analyzed online at 20 min intervals by gas chromatography equipped with a thermal conductive detector (TCD) and a 5 Å molecular sieve column, using argon as the carrier gas.

For photocatalytic oxygen evolution, 3wt% $Co(OH)_2$ was first loaded on the PDINH/$C_{60}NH_3$ as co-catalyst by immersion method [4]. Then, 7 mg catalysts were dispersed in 100 ml of 20 mM $AgNO_3$ solution and 0.1 g of La_2O_3 was added to maintain the pH. The suspension was then thoroughly degassed and irradiated with a 300 W xenon lamp with a cut-off filter ($\lambda \geq 420$ nm, 478 mW/cm^2). The apparent quantum efficiency (AQE) of oxygen evolution was measured using a 300 W xenon lamp (MC-PF300, Beijing MerryChange Technology Co.,Ltd) with bandpass filters at 420, 450, 500, 550, 600, 650 and 700 nm. The irradiation area was 1.0×1.0 cm^2.The average intensity was determined by an optical power meter (CEL-NP2000-2).

The AQE was calculated as follow equation:

$$\text{AQE} = \frac{4 \times \textit{the number of evolved } O_2 \textit{ molecules}}{N} \times 100\%$$

$$N = \frac{E\lambda}{hc}$$

N is the number of incident photons; *E* is the incident energy; λ is the wavelength of light; *h* is Planck constant (6.626×10^{-34}); *c* is speed of light (3×10^8 m/s).

In the cycling experiments, 100 mg of catalyst was used in order to avoid the effect of mass loss on the performance during the tests. After each test, the catalyst was washed with a 0.1 M HNO_3 solution to remove the Ag particles (from photoreduction of $AgNO_3$) covering on the catalyst surface.

$^{18}O_2$ labeling experiment: The catalyst was dispersed in 1 mL of $H_2{}^{18}O$ with $AgNO_3$ as the sacrificial agent. The solution was purged with Ar for 10 minutes to remove oxygen. After 30 minutes of light irradiation, 200 μL of the evolved gas was sampled using a microsyringe and injected into a GC-MS system (QP2010 ultra, Shimadzu) for analysis.

1.10 Computational methods

All the density functional theory (DFT) calculations in this work were conducted with Vienna ab initio Simulation Package (VASP) [5-7]. The electron-ion interactions were treated within the projector augmented-wave (PAW) potentials [8]. The Perdew-Burke-Ernzerhof (PBE) functional of the

generalized gradient approximation (GGA) was used to calculate the exchange and correlation interactions between electrons [9]. The cutoff energy for the plane wave basis was set as 500 eV. The long-range van der Waals (vdW) contributions in local chemical environment were evaluated with the DFT-D3 method. A criterion of 10^{-4} eV was set for energy convergence of electronic calculations, and the optimization would stop when the residual Hellmann-Feynman force on each atom was smaller than 0.01 eV/Å. The k-point sampling of the first Brillouin zone was sampled by a $2\times1\times1$ grid for geometry optimizations and electronic property computations. Molecular electrostatic potential and Transition dipole moment density analysis were analyzed and drawn by Multiwfn program [10] combined with VMD software [11].

Supplemental Figures

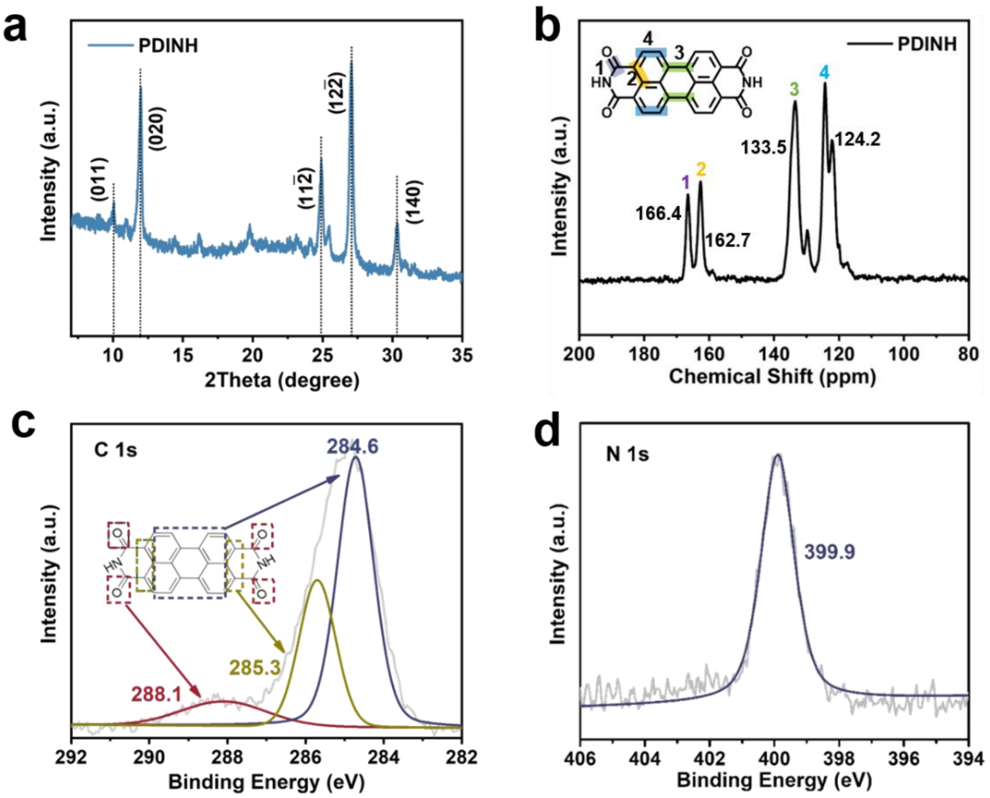

Figure S1. Structural characterizations of PDINH. (a) XRD patterns. (b) Solid ^{13}C NMR spectrum. (c) C 1s XPS spectrum. (d) N 1s XPS spectrum.

The chemical structure of PDINH was verified through XRD, solid ^{13}C NMR spectrum and XPS spectrum. As shown in **Fig. S1a**, the diffraction peaks of 27.2 ° and 12.0 ° correspond to the $(12\bar{2})$ plane and (020) plane, which are distinctive characteristic of face-to-face π-π stacking and edge-to-face π-π stacking [12]. The diffraction peak of 10.2 ° is attributed to the (011) plane, correlating to the intermolecular hydrogen bonding between the imide and carbonyl [13]. The solid ^{13}C NMR spectrum shows two peaks of 166.74 ppm and 162.74 ppm at the low field, corresponding to carbonyl carbon and

edge carbon of the perylene ring (**Fig. S1b**). Due to the high conjugation of the perylene ring, the chemical shifts of the remaining carbon atoms are difficult to assign. As observed in XPS results, the binding energies of C 1s at 284.6 eV, 285.3 eV and 288.1 eV are attributed to the inner perylene ring, edge perylene ring and carbonyl carbon, respectively (**Fig. S1c**) [1]. The XPS of N 1s shows only one peak, assigned to imine moiety (**Fig. S1d**). These results confirmed supramolecular PDINH is successfully synthesized.

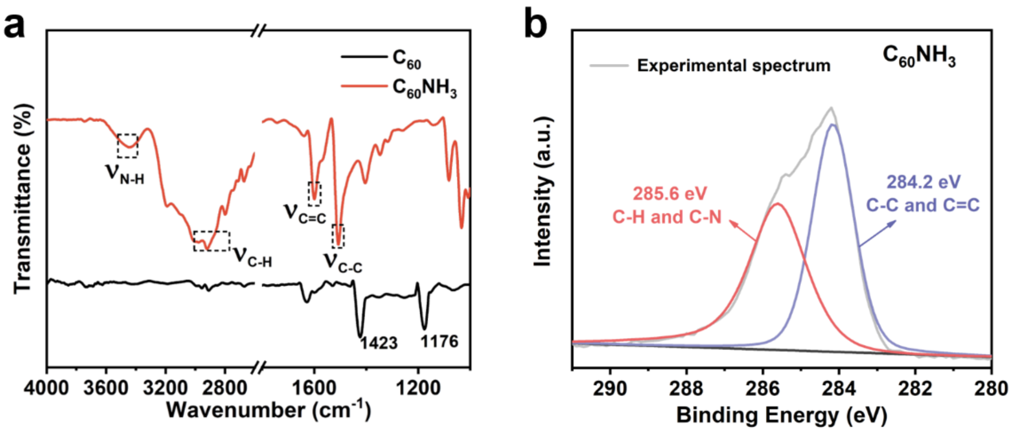

Figure S2. Structural characterizations of $C_{60}NH_3$. (a) FT-IR spectra of commercial fullerene (C_{60}) and protonated ethylenediamine-functionalized fullerene ($C_{60}NH_3$). (b) C 1s XPS spectrum of $C_{60}NH_3$.

The ethylenediamine functionalized fullerene ($C_{60}NH_3$) was prepared according to literature [2] with minor modifications, and the obtained samples were confirmed by FT-IR and XPS. As shown in **Fig. S2a**, the stretching vibrational bands of N-H and C-H are observed in $C_{60}NH_3$, but not in commercial C_{60}, indicating that ethylenediamine moieties have been bonded to C_{60}. Besides, the C 1s peaks centered at the binding energies of 284.2 eV and 285.6 eV are assigned to C-C (C=C) and C-N (C-H) for $C_{60}NH_3$, respectively [14], further confirming the successful formation of ethylenediamine functionalized fullerene (**Fig. S2b**).

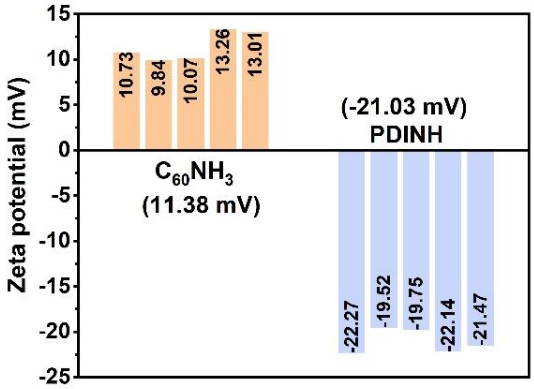

Figure S3. Zeta potentials. Zeta potentials of $C_{60}NH_3$ and PDINH at pH 7.2 (maintained with 1 mM PBS buffer) and 25 ℃. Five tests for each sample.

As shown in **Fig. S3**, the average Zeta potential of $C_{60}NH_3$ and PDINH is 11.38 mV and -21.03 mV, respectively. Therefore, the strong electrostatic attraction between positively charged $C_{60}NH_3$ and negatively charged PDINH occurs, which is favorable for the intimate integration of fullerenes on the surface of the supramolecular PDINH.

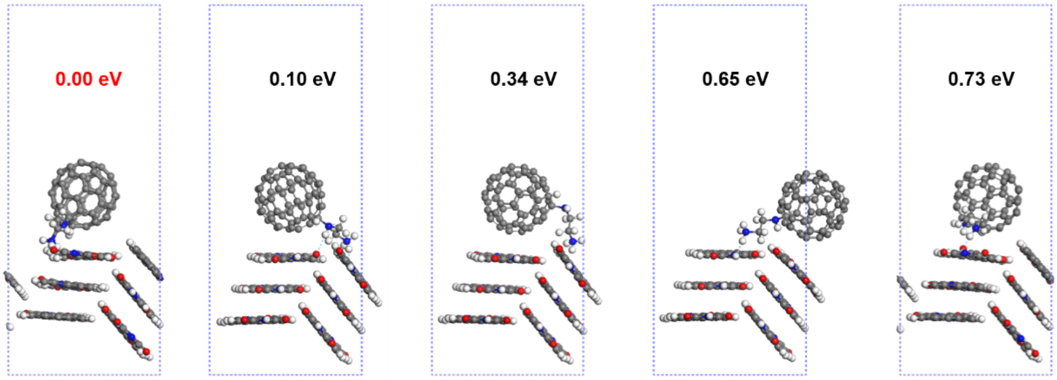

Figure S4. DFT optimized structures. DFT optimized adsorption configurations of $C_{60}NH_3$ on PDINH $(12\bar{2})$ plane and their relative energies.

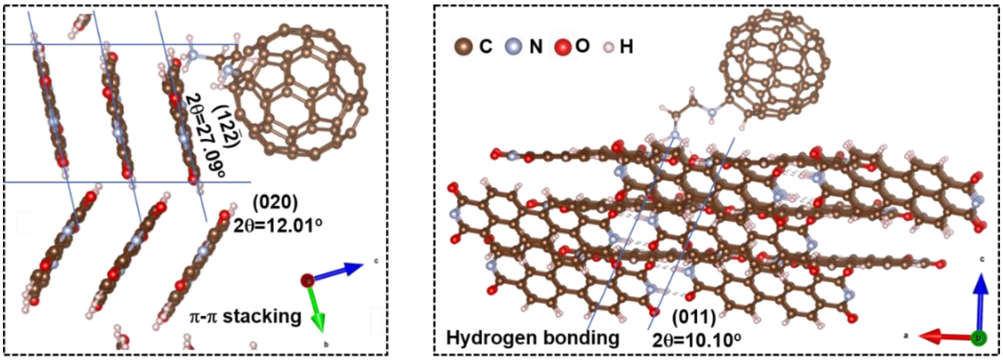

Figure S5. Geometric structure of PDINH/$C_{60}NH_3$ with different viewpoints.

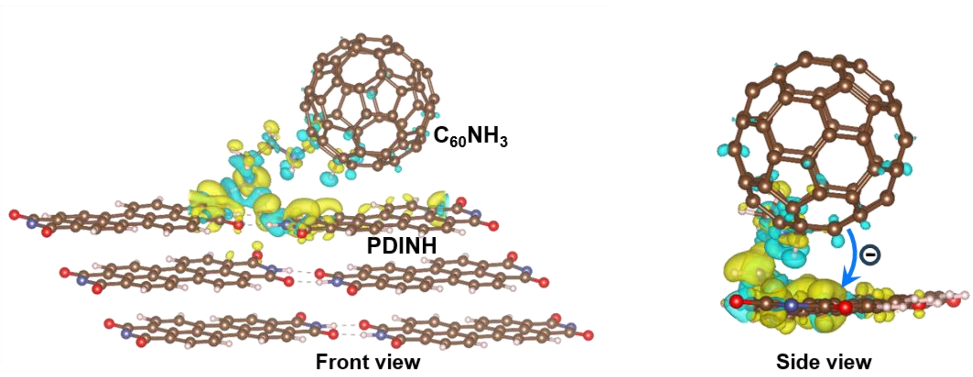

Figure S6. Charge density difference analysis.

Differential charge density of PDINH/$C_{60}NH_3$, in which the yellow and blue areas denote the electronic accumulation and depletion, respectively. Isosurface is 0.001 e/Bohr^3. The differential charge reveals the obvious correlations of C=O in PDINH and the N-H bonds in $C_{60}NH_3$. Hydrogen bonds act

as charge transfer bridges, inducing electron transfer from fullerene to PDINH, thereby modulating the potential distribution in PDINH.

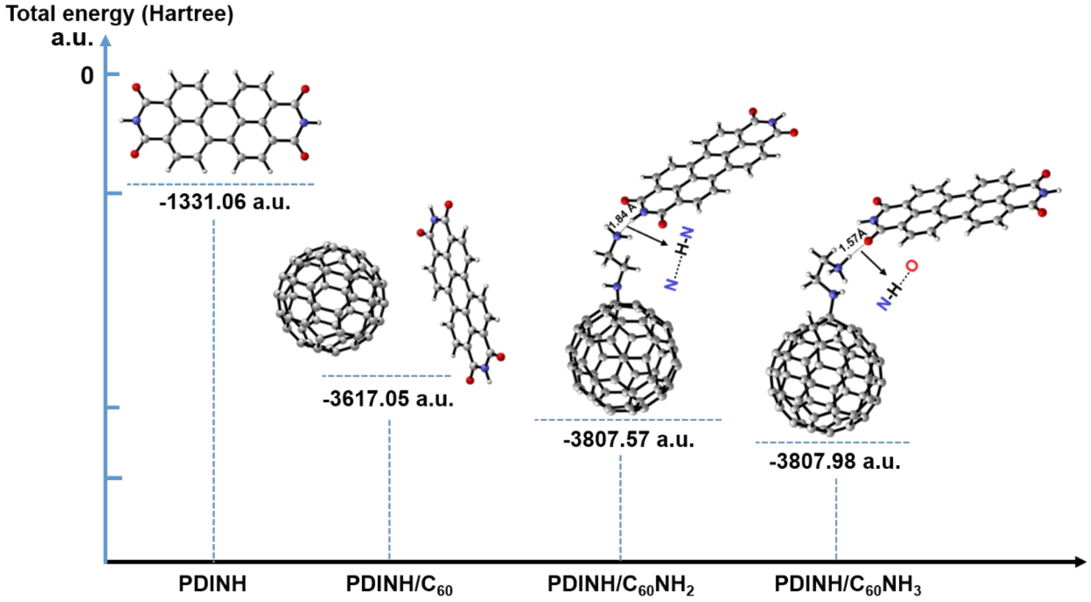

Figure S7. Hartree energy analysis by DFT. The total energies and hydrogen bonding interactions between fullerenes and PDINH by DFT calculations.

As shown in **Fig. S7**, the H atom in the N-H groups of $C_{60}NH_3$ points toward the O atom in the C=O group of PDINH, while the N atom in the N-H groups of $C_{60}NH_2$ points toward the H atom in the imide group (-CONH) of PDINH, indicating that the hydrogen bond sites can be regulated by controlling the protonation of amino-modified fullerene. Interestingly, the H···O distance in PDINH/$C_{60}NH_3$ assembly is much shorter than H···N distance (1.84 Å) in PDINH/$C_{60}NH_2$, suggesting that the H···O hydrogen bonding is stronger and thus making PDINH/$C_{60}NH_3$ more stable. The consistent energy lowering combined with characteristic structural parameters, strongly suggesting that hydrogen bonding contributes to the relative stability of these configurations.

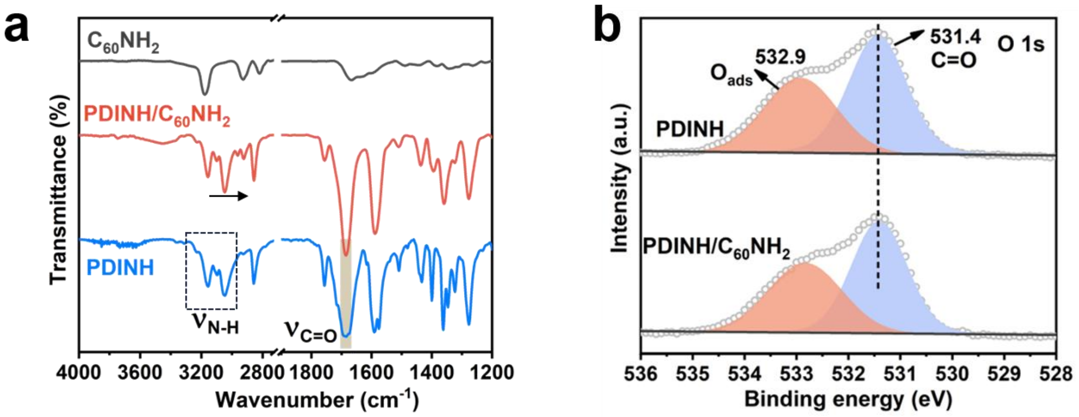

Figure S8. Structural analysis of PDINH/$C_{60}NH_2$. (a) FT-IR spectra of PDINH, $C_{60}NH_2$ and PDINH/$C_{60}NH_2$. (b) O 1s XPS spectrum of PDINH and PDINH/$C_{60}NH_2$.

As shown in **Fig. S8a**, When PDINH is assembled with $C_{60}NH_2$, the N-H stretching vibrations in the imide shifts slightly to lower wavenumbers, and stretching vibrations of C=O in PDINH has not shift after introducing $C_{60}NH_2$, indicating hydrogen bonding interactions between N atom of $C_{60}NH_2$ and imide (-CONH) of PDINH occurs. Moreover, the binding energy of O 1s peaks in PDINH is unchanged (**Fig. S8b**), further indicating the hydrogen bond site in PDINH/$C_{60}NH_2$ assembly is imide rather than carbonyl oxygen. There is no hydrogen available to form hydrogen bond between commercial C_{60} and PDINH.

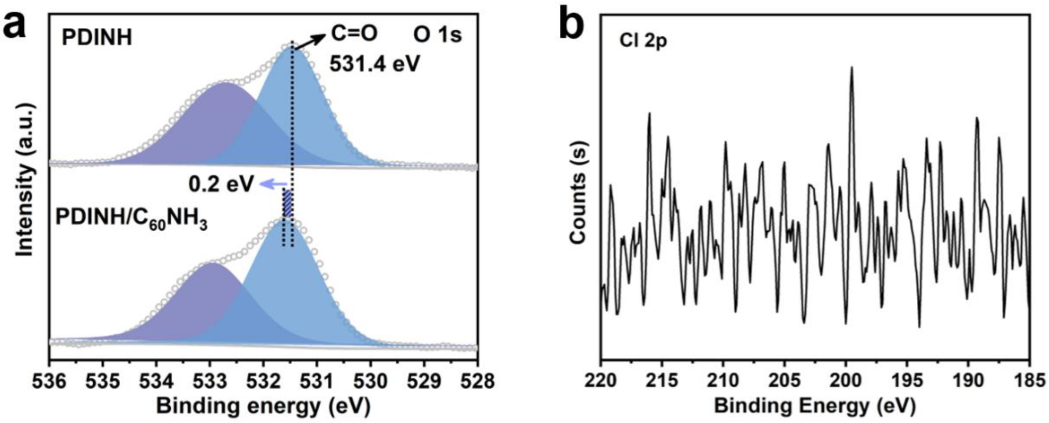

Figure S9. XPS analysis of PDINH/$C_{60}NH_3$. (a) O 1s XPS spectrum of PDINH and PDINH/$C_{60}NH_3$; (b) Cl 2p XPS spectrum of PDINH/$C_{60}NH_3$.

Typically, the N-H···O=C hydrogen bond enhances C=O bond polarization, resulting in reduced electron density at the oxygen atom. As shown in **Fig. S9a**, the binding energy of O 1s of PDINH/$C_{60}NH_3$ is higher compared to the pristine PDINH, suggesting a decrease in electron density of carbonyl oxygen because of the H···O hydrogen bond formation. The **Cl 2p** spectrum indicates that the Cl content in the final synthesized PDINH/$C_{60}NH_3$ is below the detection limit (< 0.1 at%), ruling out any Cl residue.

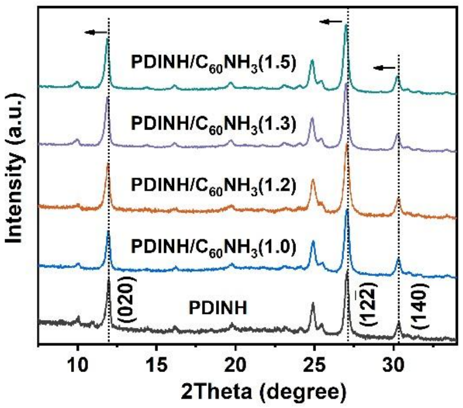

Figure S10. XRD analysis. XRD patterns of PDNH/$C_{60}NH_3$ composites with different contents of $C_{60}NH_3$ (wt%).

As shown in **Fig. S10**, the (12$\bar{2}$) plane and (020) plane correspond to the face-to-face π-π stacking and edge-to-face π-π stacking, respectively. After introducing $C_{60}NH_3$, these diffraction peaks gradually shift to lower degrees, indicating the interlayer distance between PDINH molecules becomes longer caused by the strong adsorption of $C_{60}NH_3$ on the (12$\bar{2}$) plane. Meanwhile, the (140) plane diffraction peak (oxygen-associated) also shows a slight low-angle shift. Combined with DFT-calculated bond lengths and vibrational spectroscopy changes, these observations collectively suggest N-H···O hydrogen bonding.

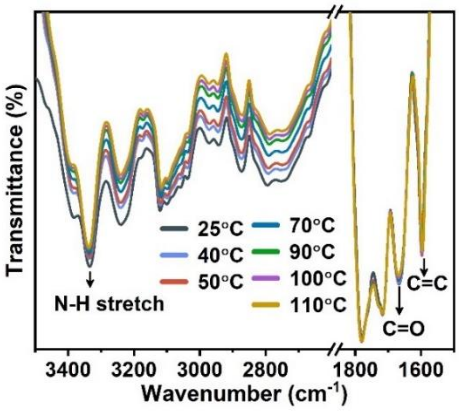

Figure S11. Temperature-dependent FT-IR spectra. IR spectra of PDINH/$C_{60}NH_3$ upon increasing the temperature from 25°C to 110°C.

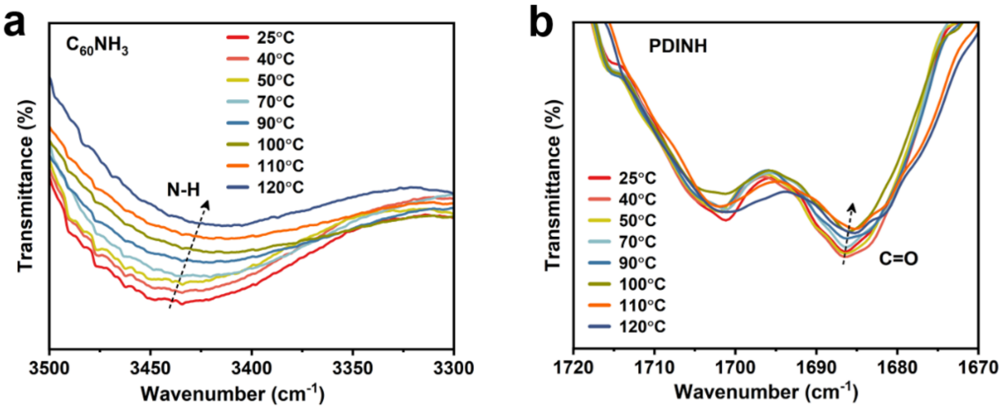

Figure S12. Temperature-dependent FT-IR spectra. IR spectra of (a) PDINH and (b) $C_{60}NH_3$ upon increasing the temperature from 25°C to 120°C.

As shown in **Fig. S11**, with the temperature increasing from 25°C to 110°C, the N-H stretch vibrations and C=O stretch vibrations are simultaneously blue-shifted, and the absorption intensity is weakened, demonstrating the existence of hydrogen bond between the carbonyl group and the amino

group [15, 16]. It is noteworthy that the IR spectra of the single-component PDINH and $C_{60}NH_3$ reveal a red-shift in both N-H vibrations and C=O vibrations with temperature elevating (**Fig. S12**). This observation is consistent with anharmonicity and provides clear evidence for the N-H···O hydrogen bond formation in PDINH/$C_{60}NH_3$.

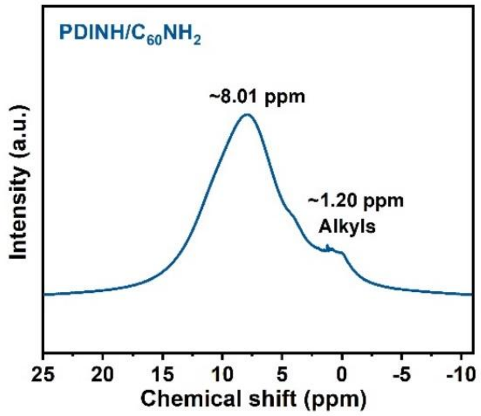

Figure S13. Solid-state ^{1}H NMR spectra of PDINH/$C_{60}NH_2$.

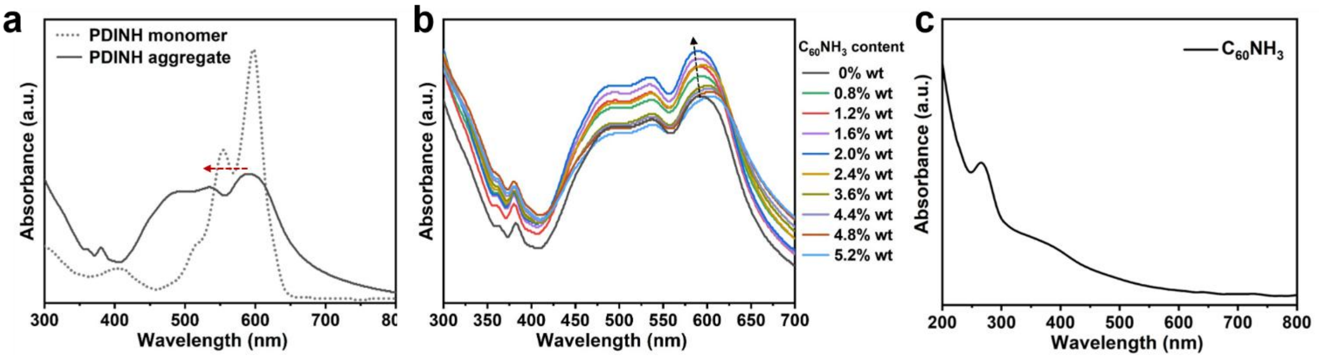

Figure S14. UV-Vis absorption spectra. (a) Supramolecular PDINH dispersed in water (2.0×10^{-5} M) (solid line) and PDINH monomer dissolved in H_2SO_4 (dashed line). PDINH/$C_{60}NH_3$ aqueous solution with different $C_{60}NH_3$ contents. (c) $C_{60}NH_3$ suspended in water.

As shown in **Fig. S14**, the maximum absorption of the supramolecular PDINH is blue-shifted relative to the PDINH monomer, which is in agreement with the H-aggregate formation [13]. The absorption is slightly blue-shifted and intensified in the visible range after anchoring of $C_{60}NH_3$ onto the supramolecular PDINH, which is coincident with the absorption spectral characteristics of the hydrogen-bonded supramolecular materials. Nevertheless, excessive amounts of $C_{60}NH_3$ (> 3.6% wt) lead to diminished and red-shifted absorption in the visible region, suggesting that π-π stacking interactions dominate the PDINH/$C_{60}NH_3$ assembly. This stacking is typical in aromatic and fullerene systems [17], where strong interactions between π-electron at close distances cause electron delocalization, lowering the transition energy and inducing a red-shift.

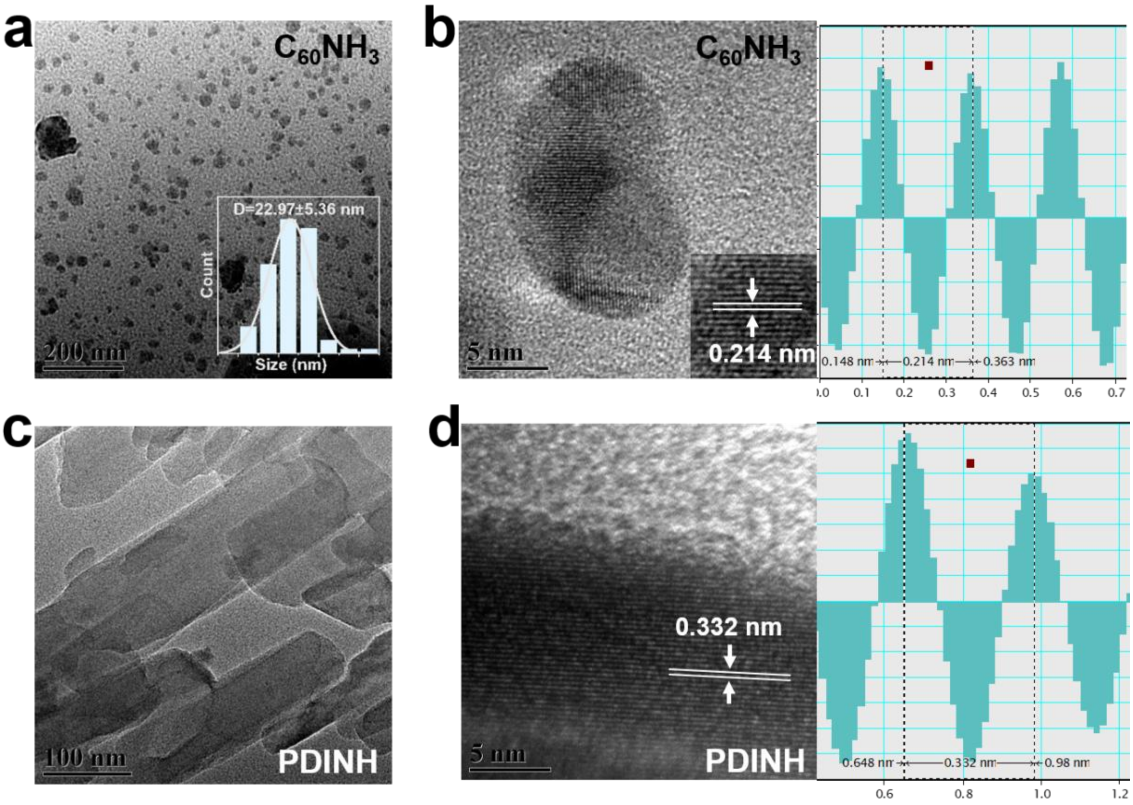

Figure S15. Morphological characterizations of $C_{60}NH_3$ and PDINH. (a) TEM and (b) HRTEM images of $C_{60}NH_3$. (c) TEM and (d) HRTEM images of PDINH.

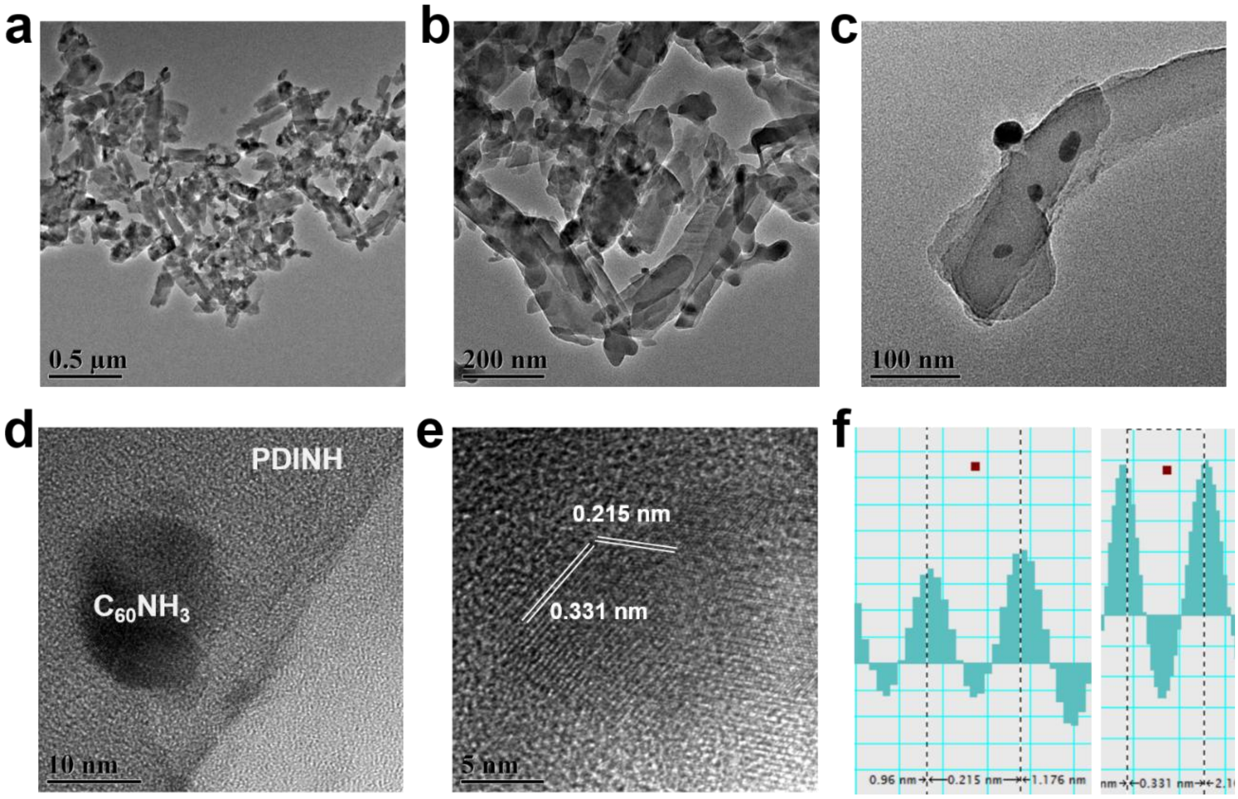

Figure S16. Morphological characterizations of PDINH/$C_{60}NH_3$. (a-c) TEM images of PDINH/$C_{60}NH_3$. (d-f) HRTEM images of PDINH/$C_{60}NH_3$.

As shown in **Fig. S15a** and **S15b,** $C_{60}NH_3$ presents nanoparticles with a statistical average size of 22.97 nm, and a characteristic lattice fringe of 0.214 nm is also observed. The supramolecular PDINH shows a quasi-hexagonal morphology within a micrometer scale in lateral dimension (**Fig. S15c** and **S15d**), which is consistent with the previous report [1]. It can be clearly observed that $C_{60}NH_3$ nanoparticles are distributed on the surface of PDINH (**Fig. S16**), indicating the successful construction of hydrogen-bond-linked PDINH/$C_{60}NH_3$ assembly.

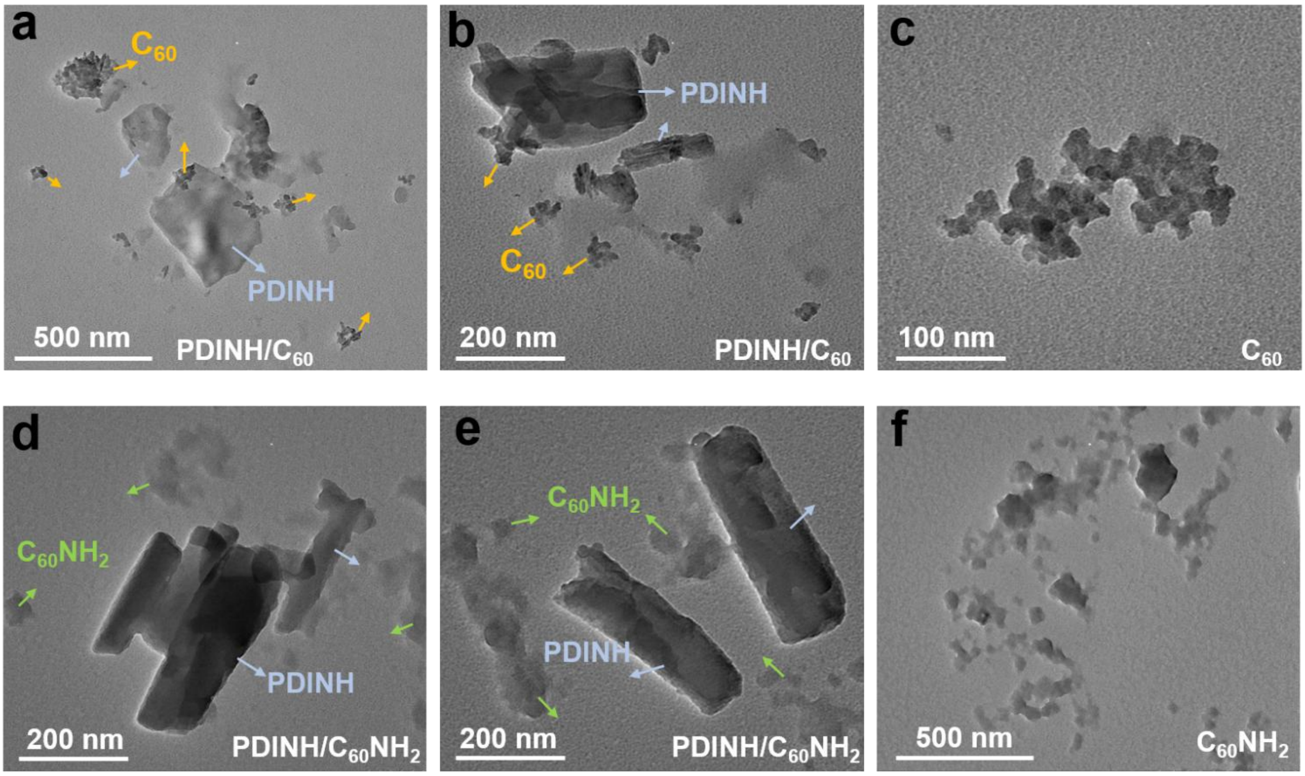

Figure S17. TEM characterizations. (a-c) TEM images of PDINH/C_{60}. (d-f) TEM images of PDINH/$C_{60}NH_2$.

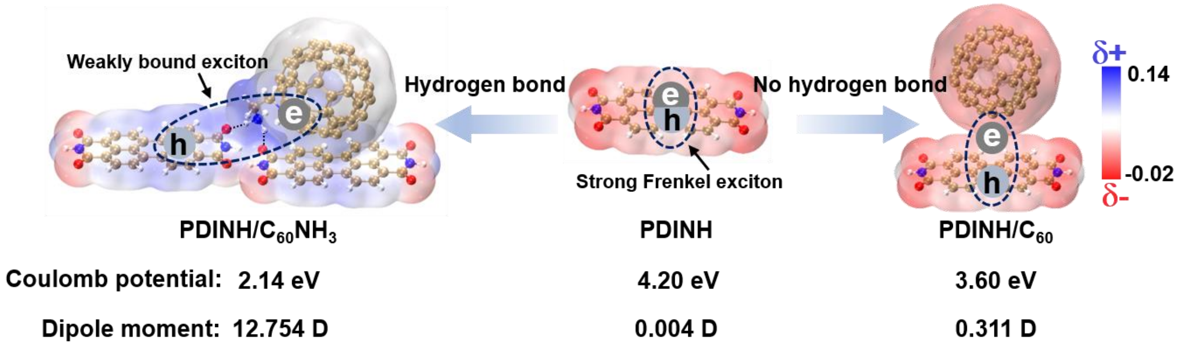

Figure S18. DFT calculated molecular electrostatic potentials (ESP) distribution of the ground state, Coulomb interaction potential of electron-hole pairs and the dipole moment of PDINH, $C_{60}NH_3$ and PDINH/$C_{60}NH_3$.

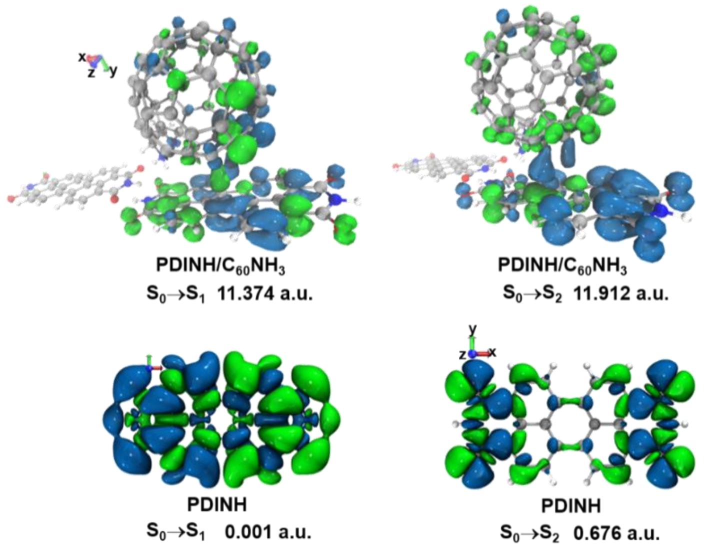

Figure S19. TD-DFT calculated excited states of PDINH and PDINH/$C_{60}NH_3$. The excited states of PDINH and PDINH/$C_{60}NH_3$ and the corresponding transition dipole moment. Blue regions and green regions represent positive and negative, respectively, the isosurface value is 0.001 a.u.

As shown in **Fig. S19**, the hydrogen-bond-linked PDINH/$C_{60}NH_3$ enhances exciton delocalization, where the positive (holes) charge density is mainly concentrated on the perylene core and negative (electrons) charge density on the fullerenes. This reduces Coulombic attraction of electron-hole pairs and thus promoting exciton dissociation.

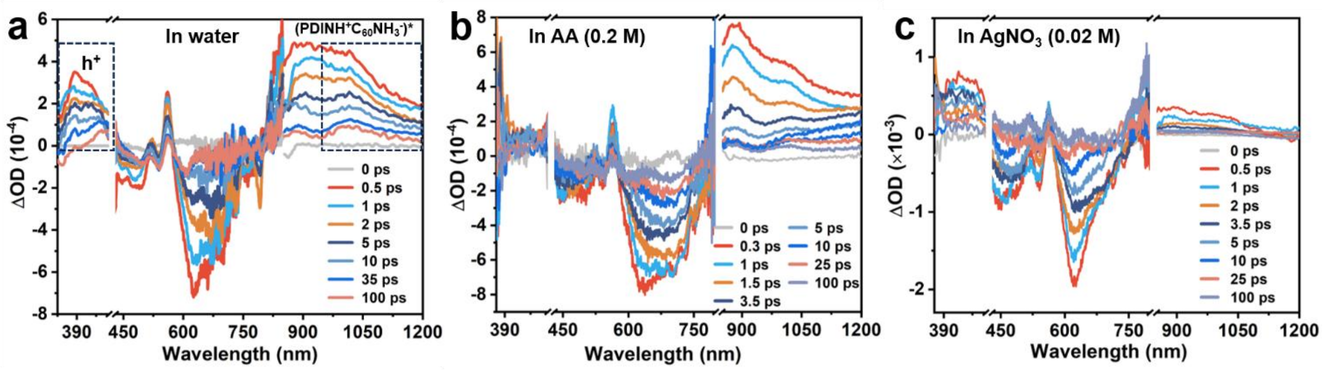

Figure S20. fs-TA spectra of PDINH/$C_{60}NH_3$ in different solvents at 420 nm photoexcitation.

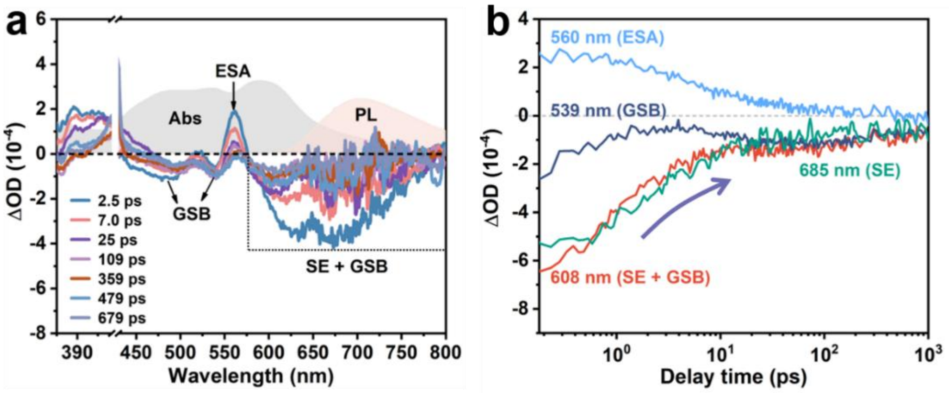

Figure S21. fs-TA spectra of PDINH/$C_{60}NH_3$ with (a) the steady-state absorption spectra (Abs, grey shadow) and photoluminescence emission spectra (PL, pink shadow) and (b) Temporal profiles of the time-resolved absorption signal at selected wavelengths.

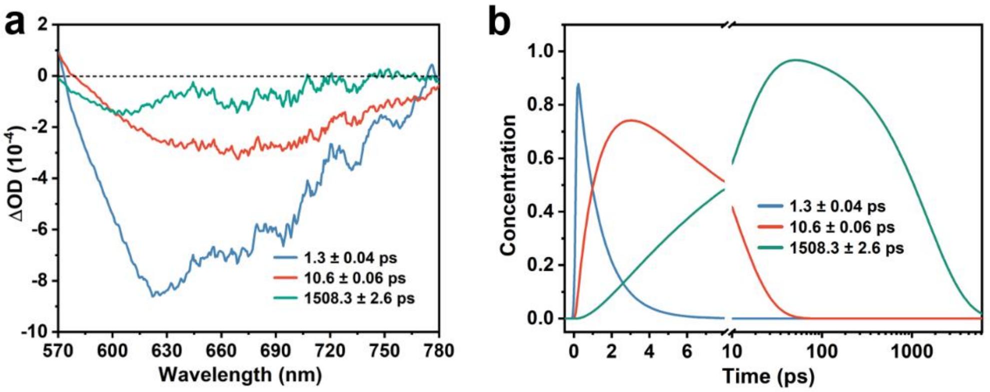

Figure S22. Global analysis of the TA data at 570-780 nm. (a) Species-associated spectra plots from global analysis. **(b)** Relative population profiles with the dynamics of transient species for PDINH/$C_{60}NH_3$ from global analysis.

The negative signal at 600-700 nm can be attributed to the spectral overlap between ground-state bleaching (GSB) and stimulated emission (SE), as evidenced by its coincidence with both the ground-state absorption and fluorescence emission bands (Fig. S21). As shown in Fig. S22, the global analysis of TA data (570-780 nm) reveals three distinct kinetic components: A fast 1.3 ps component corresponding to vibrational relaxation in S_1 state of PDINH [18], consistent with the initial spectral narrowing observed in this region; The second component of ~10.6 ps corresponds to radiative transition of S_1, confirming its SE origin; A slow 1508.3 ps component reflects long-lived GSB due to ground-state repopulation. The prolonged GSB recovery (>1 ns) suggests stabilization through PDINH-fullerene charge-transfer state formation, which significantly retards charge recombination [19]. These results provide definitive evidence for the GSB and SE overlapping in the 600-700 nm region.

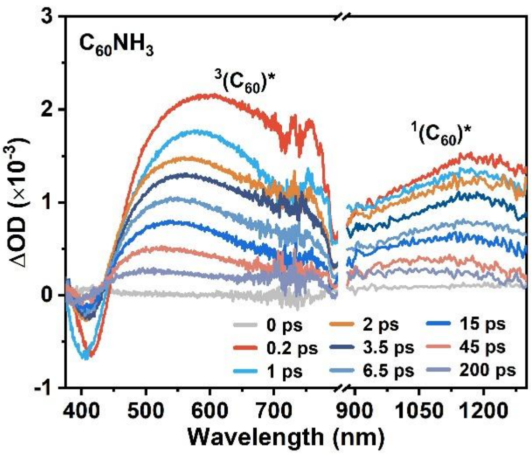

Figure S23. fs-TA spectra of $C_{60}NH_3$ at 420 nm photoexcitation.

The fs-TA spectrum of $C_{60}NH_3$ was further measured to validate TA signal assignment. The two distinct positive ESA bands at 500-750 nm and 900-1200 nm correspond to characteristic absorption of triplet $^3(C_{60})^*$ and singlet $^1(C_{60})^*$ states, respectively. Notably, the functionalized-C_{60} induces significant blue-shifts in these ESA bands compared to pristine C_{60} due to the perturbation of C=C double bonds [20]. The TA signal of $C_{60}NH_3$ alone in the 600-700 nm range differs from that of PDINH/$C_{60}NH_3$, further supporting the spectral overlap between GSB and SE.

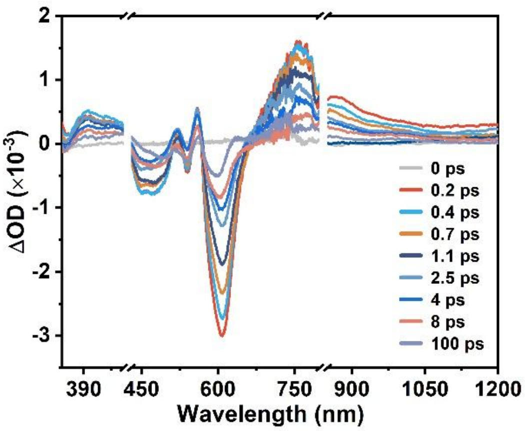

Figure S24. fs-TA spectra of PDINH/C_{60} at 420 nm photoexcitation.

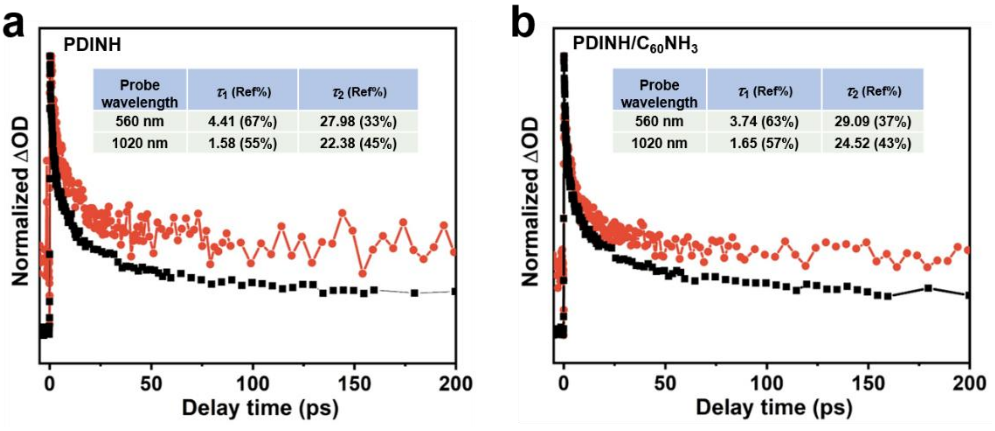

Figure S25. Transient absorption decay curves. The normalized TA kinetics at 560 nm and 1020 nm of (a) PDINH/$C_{60}NH_3$ and (b) PDINH.

The ESA at 560 nm is attributed to polarized exciton state $(PDINH^+PDINH^-)^*$ due to the electron transition of $S_1 \rightarrow S_N$ between PDINH moieties. The ESA signal at about 1020 nm is assigned to charge-transfer exciton $(PDINH^+C_{60}NH_3^-)^*$ [21]. As shown in **Fig. S25**, compared to PDINH, the decay kinetics of PDINH/$C_{60}NH_3$ is significantly faster at 560 nm absorption, while the decay kinetics becomes more sluggish at 1020 nm, indicating that $(PDINH^+PDINH^-)^*$ excitons are effectively transformed into charge-transfer excitons in PDINH/$C_{60}NH_3$. Since the charge-transfer excitons exhibit weaker binding energy, they dissociate more easily into free charge carries, thus improving charge separation efficiency.

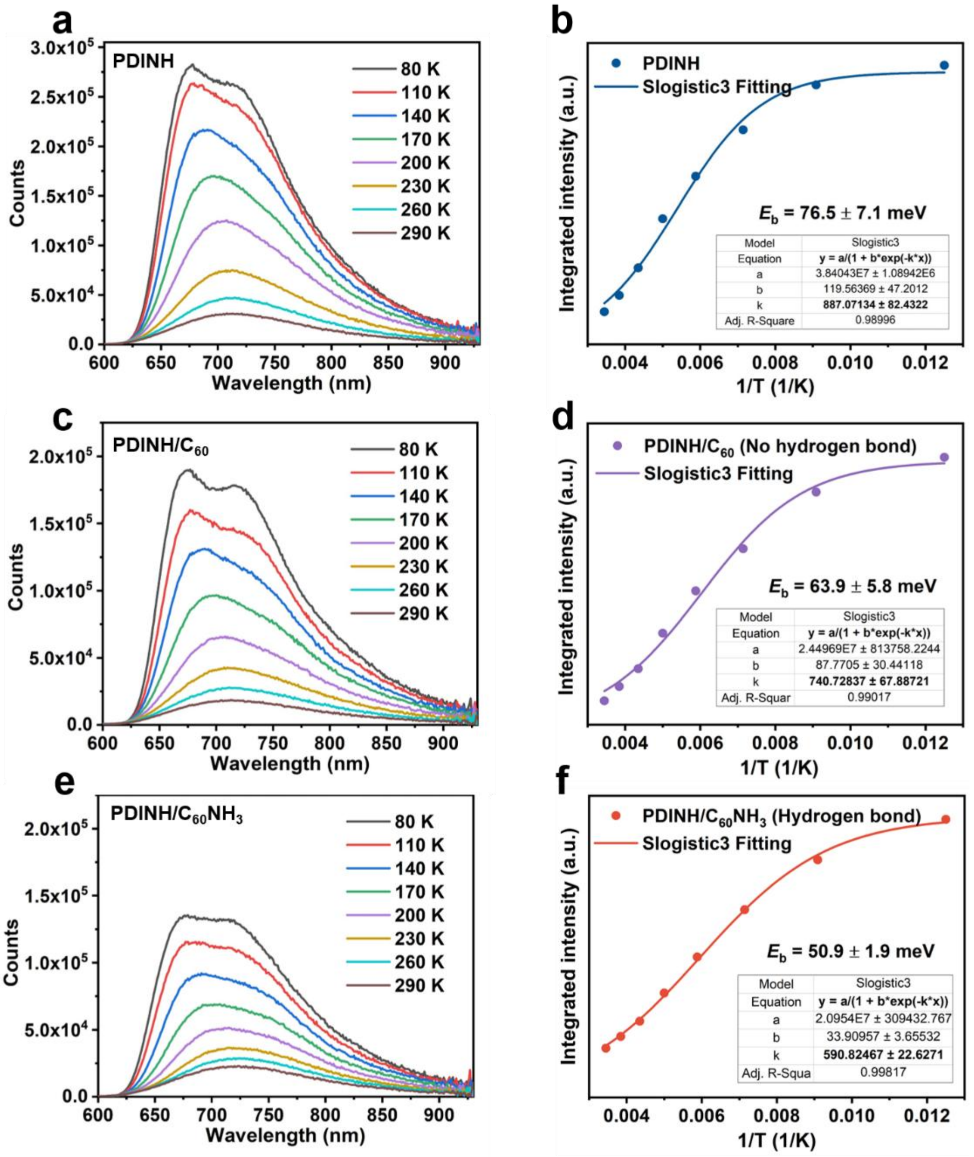

Figure S26. Temperature-dependent PL spectra under 403 nm excitation and the corresponding fitting curves. The temperature-dependent PL spectra from 80 to 290 K of (a) PDINH, (c) PDINH/C_{60} and (e) PDINH/$C_{60}NH_3$. The fitted curves of the integrated PL emission intensity as a function of 1/T for (b) PDINH, (d) PDINH/C_{60} and (f) PDINH/$C_{60}NH_3$.

The exciton binding energy (E_b) values were obtained by fitting the temperature-varying PL intensities [22, 23], $I(T)$, with the following Arrhenius equation:

$$I(T) = I_0/(1 + Ae^{-E_b/k_B T})$$

Where I_0 is the PL intensity at 0 K, T is the temperature, A is a constant, k_B is the Boltzmann constant and E_b is the exciton binding energy.

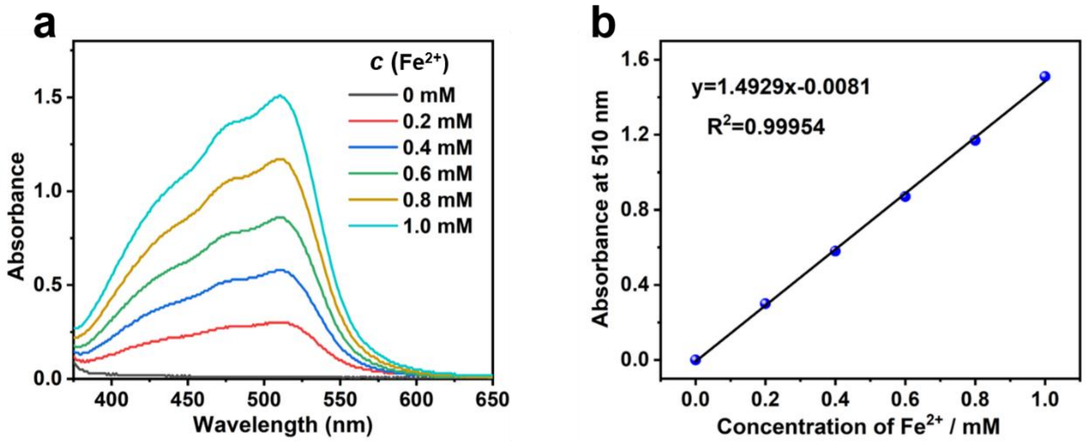

Figure S27. Calibration curve for quantitative analysis of Fe^{2+}. (a) UV-Vis absorption spectrum of Fe^{2+}. (b) Calibration curve for quantitative analysis of Fe^{2+}.

The separation efficiency of photogenerated electron-hole pairs was determined by measuring the apparent quantum efficiency (AQE) of photocatalytic reactions in the presence of electron/hole scavengers [24, 25]. Herein, we use Fe^{3+} as electron scavenger and CH_3OH as hole scavenger due to the fast kinetics of Fe^{3+} reduction and CH_3OH oxidation (relative to water oxidation). Therefore, the sluggish water oxidation reaction was replaced by a more kinetic feasible oxidation reaction of CH_3OH, this can minimize the effect of surface catalytic kinetics on the charge separation. Thus, the measured AQE can be approximately equated to the electron-hole separation efficiency. The AQE measurement is similar to photocatalytic oxygen evolution experiments. Band-pass filters with λ at 420 nm was used. The AQE (ϕ) was calculated according to the following equation:

$$\phi\,(\%) = \frac{A \times R}{I} \times 100$$

where A, R, and I represent a coefficient (1 for the reduction of Fe^{3+}), the number of reduced Fe^{3+} in the initial 10 min irradiation, and the absorbed incident photons, respectively. The total number of incident photons at λ=420 nm was measured to be 5.19×10^{19}. The number of reduced Fe^{3+} is equal to the concentration of Fe^{2+}, which can be quantified by a phenanthroline method. **Fig. S27** shows the calibration curve for quantitative analysis of Fe^{2+}.

The solution after reaction was diluted that the concentration of Fe^{2+} is less than 1.0 mM. Then 1.0 mL diluted solution was mixed with 4.0 mL NaAc-HAc buffer solution (pH=4.0) and 3.0 mL 0.01 M 1,10-phenanthroline solution. The UV-Vis absorption spectrum was measured. Then the concentration of Fe^{2+} was estimated based on the absorbance at 510 nm using a calibration curve (**Fig. S28**).

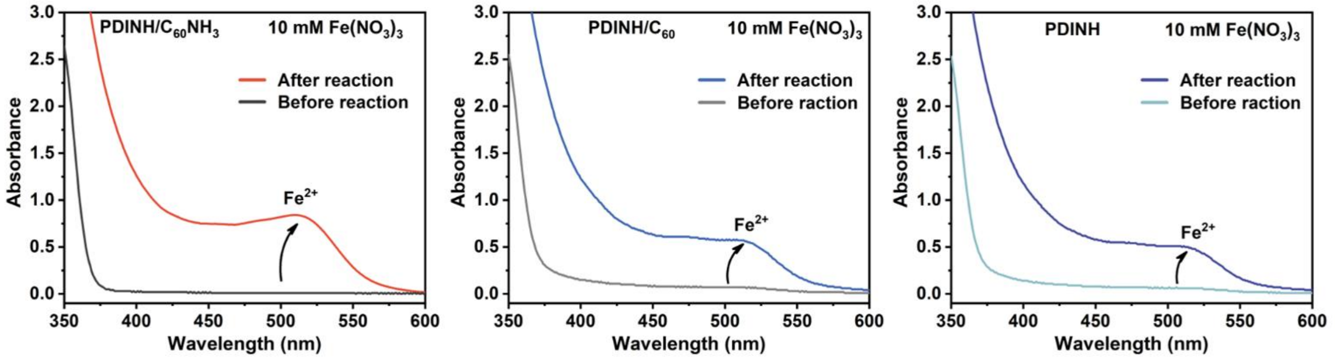

Figure S28. UV-Vis spectra of Fe^{2+} in the reaction solution before and after the reaction. Reaction condition: 10 mg catalyst; 10 mM $Fe(NO_3)_3$, 20 mL CH_3OH and 80 mL deionized water; light source, Xe lamp (300 W) with a 420 nm filter. The solution before and after reaction were diluted 10 times, and then detected by the phenanthroline method.

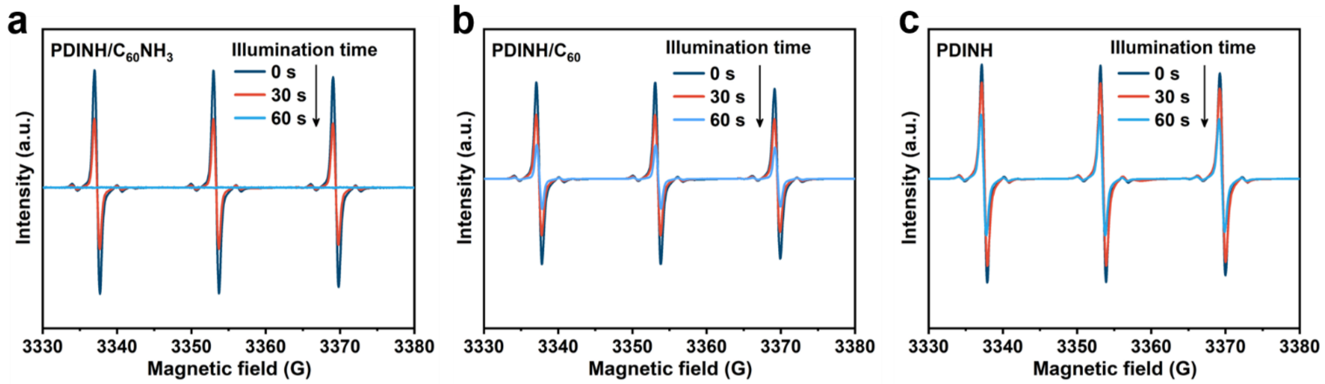

Figure S29. EPR detection of photogenerated holes by using 2,2,6,6-tetramethylpiperidine-1-oxyl/acetonitrile (TEMPO/ACN). The EPR signals under dark and upon light irradiation of (a) PDINH/$C_{60}NH_3$ (b) PDINH/C_{60} and (c) PDINH.

As shown in **Fig. S29**, TEMPO radical exhibits a characteristic triplet EPR signal under dark. Upon illumination, the EPR signal intensity of TEMPO decreases owing to the oxidation of TEMPO by photogenerated holes. The faster signal decay rate directly correlates with higher concentrations of photogenerated holes. Notably, the EPR signal of PDINH/$C_{60}NH_3$ decreases substantially after 30 s of illumination and vanishes completely within 60 s, whereas PDINH and PDINH/C_{60} exhibit significantly slower EPR signal decay under the same conditions. This rapid and complete signal quenching further confirms the higher concentration of free carriers in PDINH/$C_{60}NH_3$.

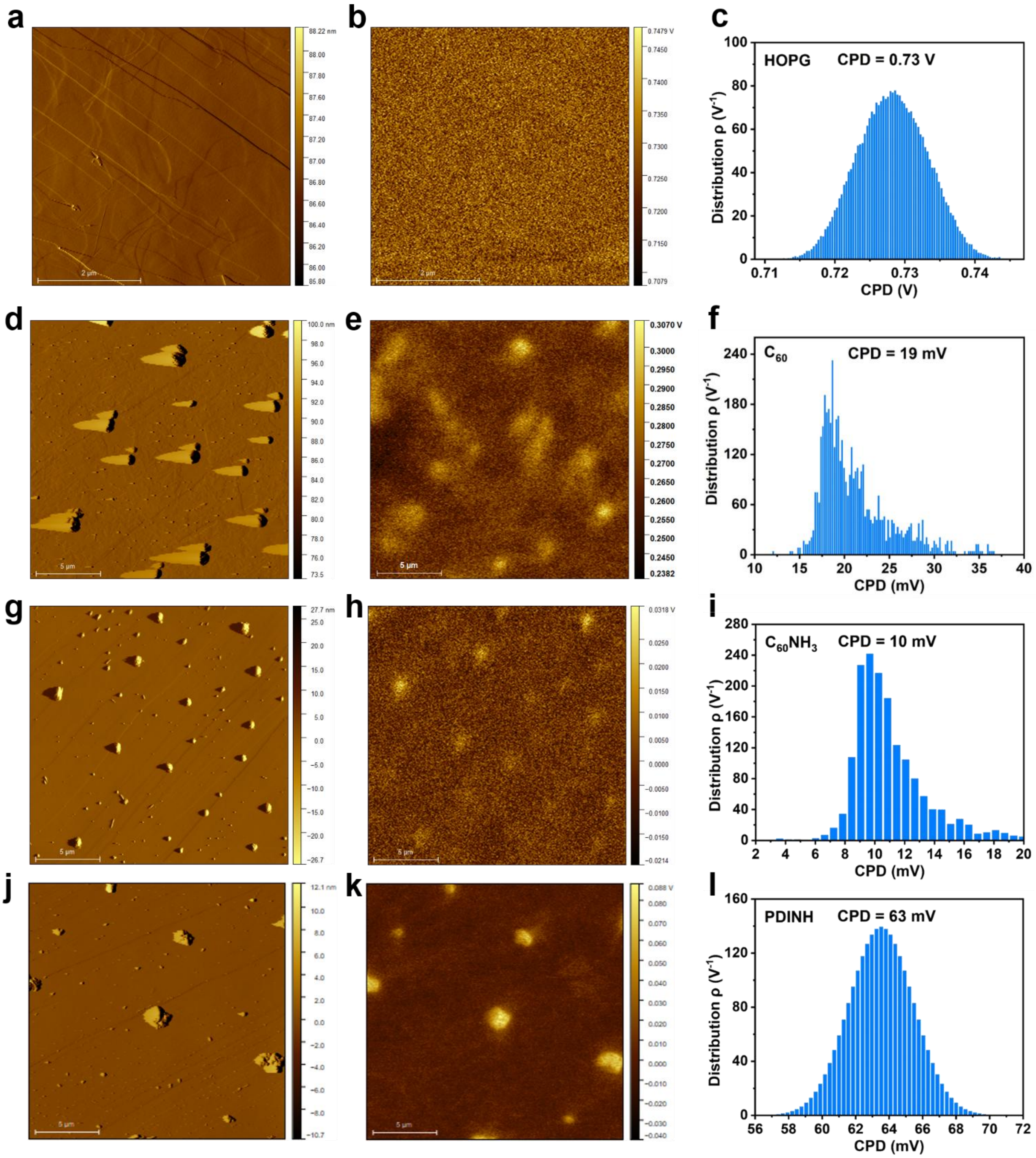

Figure S30. Contact potential difference (CPD) between probe and samples measured by KPFM under argon atmosphere. Morphology characterization of (a) HOPG, (d) C_{60}, (g) $C_{60}NH_3$ and (j) PDINH. The potential difference maps of (b) HOPG, (e) C_{60}, (h) $C_{60}NH_3$ and (k) PDINH. The statistical average CPD value of (c) HOPG, (f) C_{60}, (i) $C_{60}NH_3$ and (l) PDINH.

The work functions (Φ) are determined by measuring the contact potential difference (CPD) between the sample and probe using in-situ Kelvin probe force microscope (KPFM). The calculation formula is: $\Phi_{sample} = \Phi_{tip} + V_{CPD} \times e$, where e is the charge of an electron and Φ_{tip} is the work function of

the corrected probe [26]. Herein, the fresh highly oriented pyrolytic graphite (HOPG) was employed as the substrate and the HQ NSC18/Pt as probe. First, the work function of the probe was corrected according to formula $\Phi_{tip} = \Phi_{HOPG} - V_{CPD}\,e$, Φ_{HOPG} is 4.6 eV and V_{CPD} is 0.73 V (**Fig. S30a-S30c**), therefore, the corrected Φ_{tip} is calculated to be 3.87 eV. Based on the CPDs of 63 mV, 10 mV and 19 mV for PDINH, $C_{60}NH_3$ and C_{60}, respectively (**Fig. S30d-S30l**), the work functions of PDINH, $C_{60}NH_3$ and C_{60} can be determined as 3.93 eV, 3.88 eV and 3.89 eV, respectively. Compared with PDINH, the work functions of $C_{60}NH_3$ and C_{60} are both smaller, suggesting that it is easier for the electrons to escape from $C_{60}NH_3$ and C_{60}. When PDINH is in close contact with $C_{60}NH_3$ or C_{60}, electrons will spontaneously transfer from $C_{60}NH_3$ or C_{60} to PDINH at the heterogeneous interface until their Fermi levels reach equilibrium. As a result, the Fermi level of PDINH will be raised, meaning the work function of PDINH/$C_{60}NH_3$ becomes smaller compared with pure PDINH , which is well consistent with the theoretically calculated work functions (**Fig. S31**).

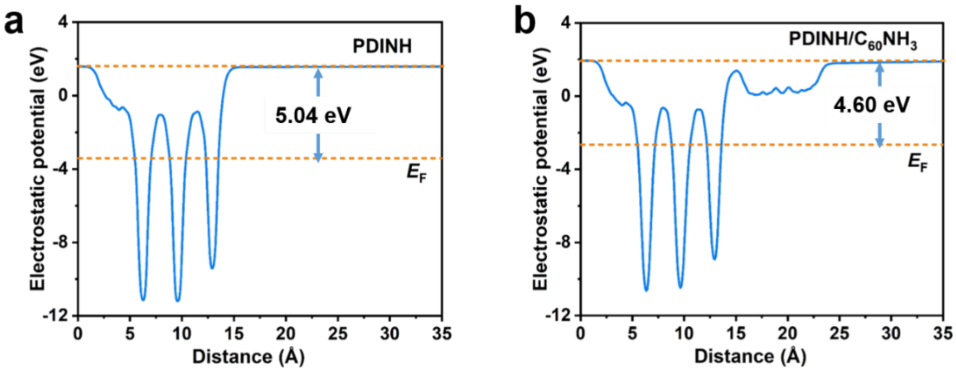

Figure S31. The DFT calculated work functions of PDINH and PDINH/$C_{60}NH_3$. The electrostatic potentials of (a) PDINH and (b) PDINH/$C_{60}NH_3$.

As shown in **Fig. S31**, the work function of the integrated system has also been explored through DFT calculation. The work function of the pure PDINH at $(12\bar{2})$ facet is calculated to be 5.04 eV. A considerably reduced work function (4.60 eV) has been identified for PDINH/$C_{60}NH_3$, which agrees well with the trend of the experimental tests (**Fig. S30**). These results suggest a change in the electrostatic potential with the introduction of $C_{60}NH_3$, further revealing an intrinsic electron transfer from $C_{60}NH_3$ to PDINH.

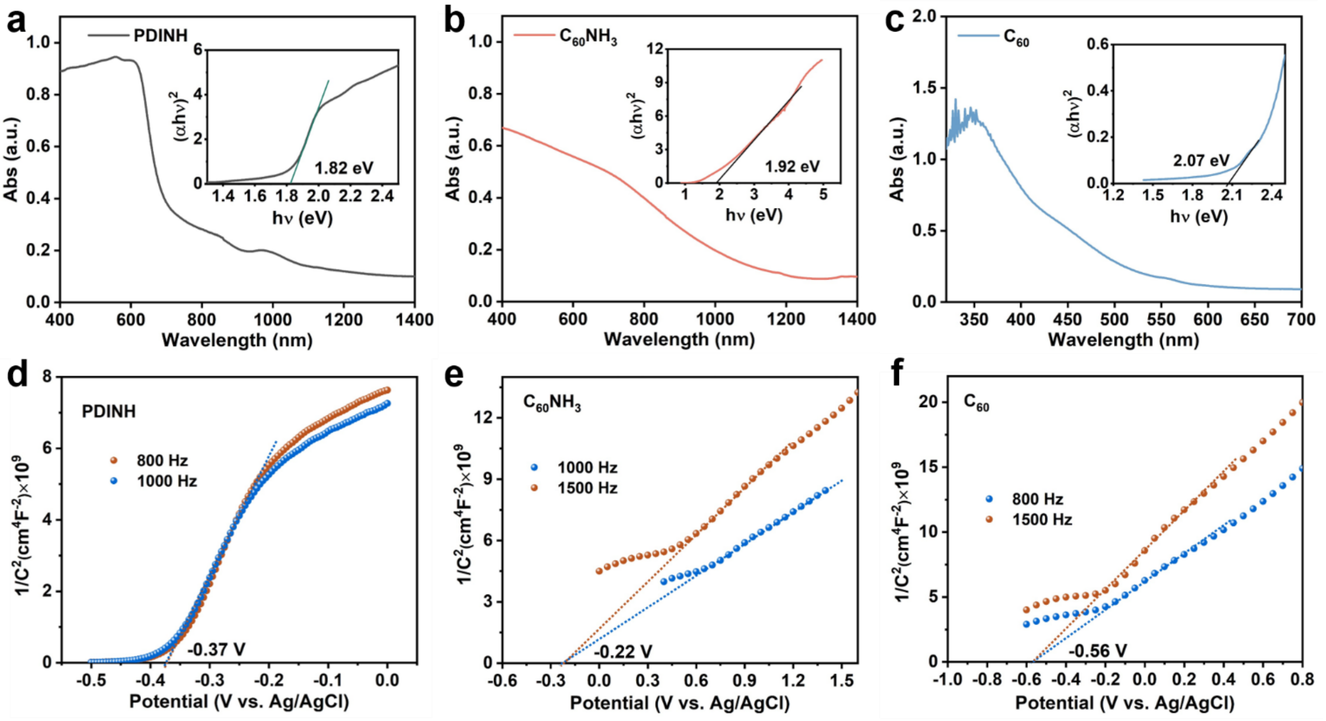

Figure S32. Energy level measurements. The UV-Vis-NIR DRS spectrum (Inset is Tauc plot) of (a) PDINH, (b) $C_{60}NH_3$ and (c) C_{60}. The Mott-Schottky plots of (d) PDINH, (e) $C_{60}NH_3$ and (f) C_{60}.

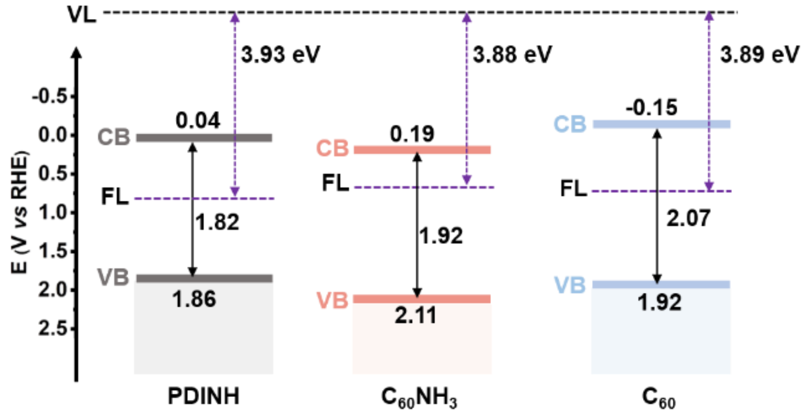

Figure S33. Energy level alignment between PDINH and $C_{60}NH_3$, C_{60}. VL is the vacuum level, CB is the conduction band minimum level, VB is the valance band maximum level and FL is the Fermi level.

As shown in **Fig. S32a-S32c**, the bandgap (E_g) can be estimated by Tauc plot from UV-Vis-NIR DRS spectrum, and the E_g are determined to be 1.82 eV, 1.92 eV and 2.07 eV for PDINH, $C_{60}NH_3$ and C_{60}, respectively. In addition, the flat band potentials of PDINH, $C_{60}NH_3$ and C_{60} are -0.37 V, -0.22 V and -0.56 V (*vs* Ag/AgCl, pH=7), respectively (**Fig. S32d-S32f**). Generally, the conduction band minimum level (CB) of an n-type semiconductor is more negative than the flat band potential by 0.1-0.2 eV. Herein, we set the voltage difference between the bottom of CB and the flat band potential to be 0.2 eV, therefore, the CB minimum level of PDINH, $C_{60}NH_3$ and C_{60} are estimated to be 0.04 V, 0.19 V

and -0.15 V (*vs* RHE), respectively, according to the equation: E_{CB} (RHE) = E_{FB} (Ag/AgCl, pH=7) +0.198+0.41. Therefore, combined E_g and CB values, the valance band maximum level (VB) is determined to be 1.86 V, 2.11 V and 1.92 V for PDINH, $C_{60}NH_3$ and C_{60}, respectively, and the energy level alignments are depicted in **Fig. S33**. From the calculated arrangement of energy levels, we found that the smallest energy mismatch (0.15 eV) is realized between PDINH and $C_{60}NH_3$, while that between PDINH and C_{60} is over 0.34 eV. The band arrangement of PDINH and $C_{60}NH_3$ is appropriate for receiving high-efficiency photoexcited electron transfer from PDINH to $C_{60}NH_3$ with minimum energy loss.

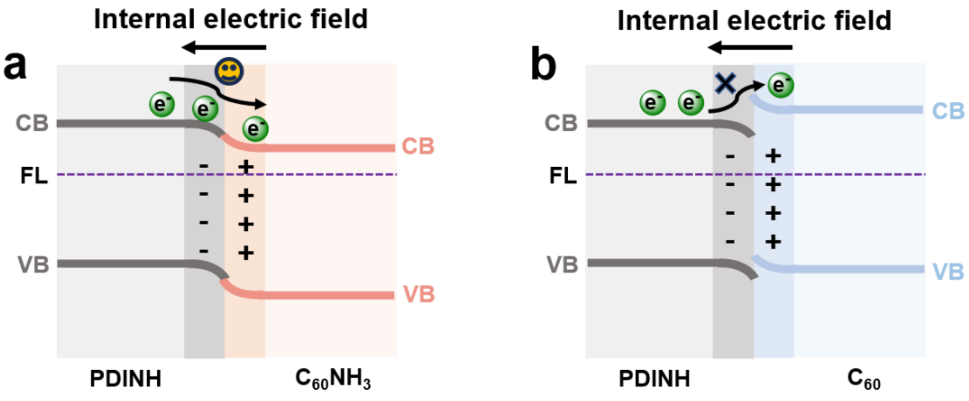

Figure S34. Band diagrams of the PDINH/$C_{60}NH_3$ and PDINH/C_{60} contacts.

The band alignments via Fermi level pinning are shown in **Fig. S34**. Since the Fermi level of $C_{60}NH_3$ is higher than that of PDINH before contact (**Fig. S33**), when PDINH and $C_{60}NH_3$ are in intimate contact, electrons transfer spontaneously from $C_{60}NH_3$ to PDINH through the heterointerface until their Fermi levels reach equilibrium. As a result, an internal electric field directed from $C_{60}NH_3$ to PDINH is generated, and the energy bands of $C_{60}NH_3$ and PDINH bend upward and downward. Notably, the PDINH/$C_{60}NH_3$ contacts can form a type II band alignment with the minimal energy-level mismatch, whereas the PDINH/C_{60} contacts cause undesired mismatch. Therefore, under the strong drive force of internal electric field, photoexcited electrons at the CB of PDINH would transfer through the interface to the CB of $C_{60}NH_3$ under light irradiation, significantly improving the separation efficiency of electrons and holes.

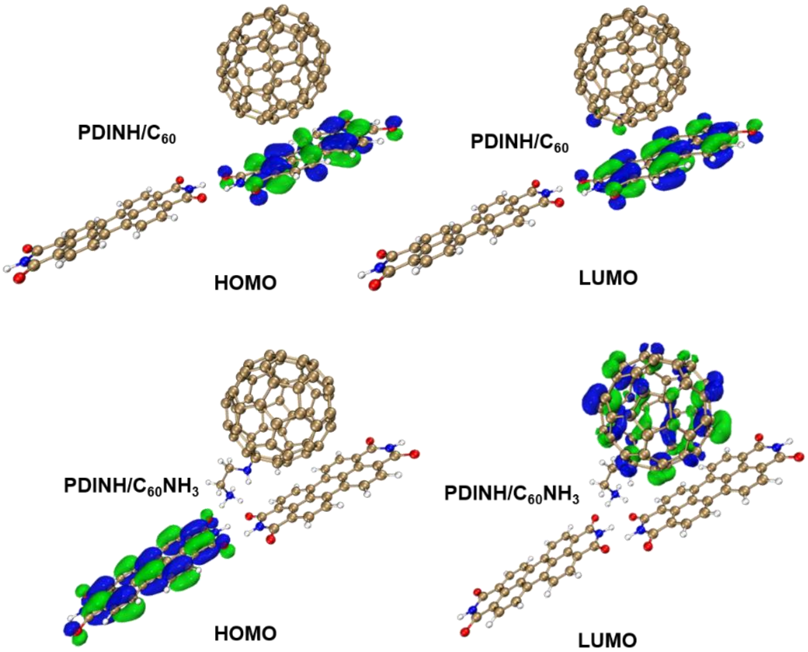

Figure S35. DFT calculated frontier molecular orbitals of PDINH/$C_{60}NH_3$ and PDINH/C_{60}.

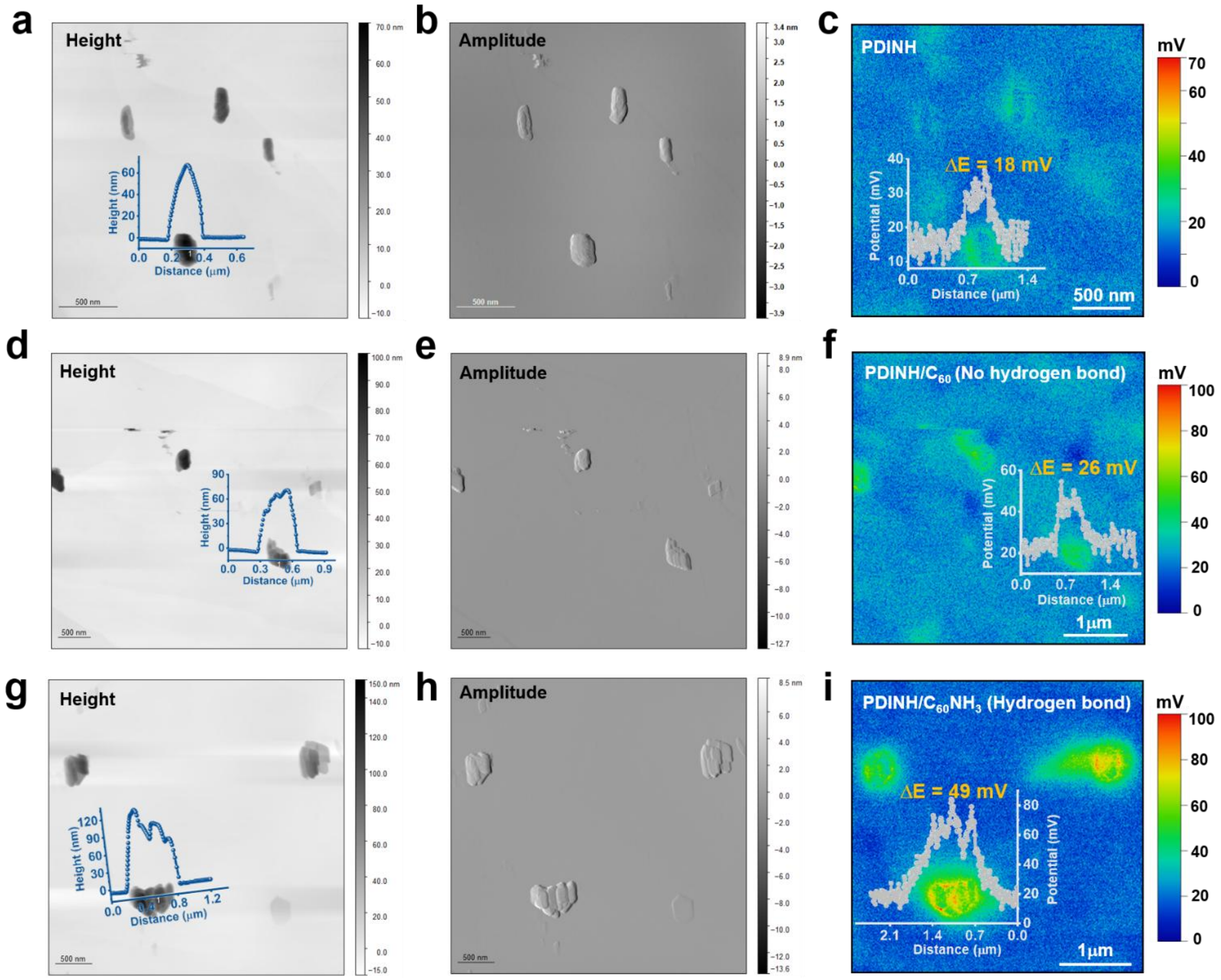

Figure S36. Surface potentials of PDINH, PDINH/C_{60} and PDINH/$C_{60}NH_3$ by KPFM under dark. KPFM images at the height mode of (a) PDINH, (d) PDINH/C_{60} and (g) PDINH/$C_{60}NH_3$. KPFM images at the amplitude mode of (b) PDINH, (e) PDINH/C_{60} and (h) PDINH/$C_{60}NH_3$. KPFM images at the surface potential mode of (c) PDINH, (f) PDINH/C_{60} and (i) PDINH/$C_{60}NH_3$.

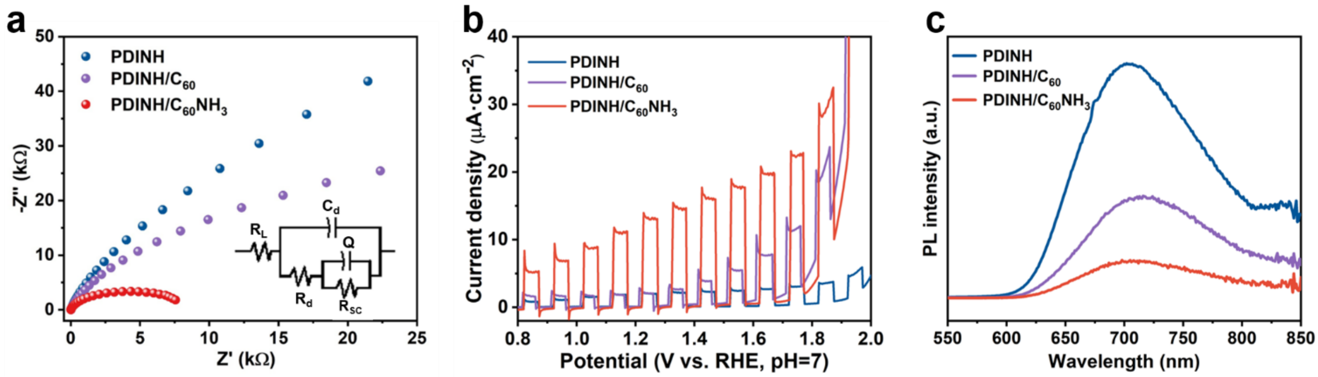

Figure S37. Characterizations of charge separation and transfer. (a) The electrochemical impedance spectroscopy (EIS) of PDINH, PDINH/C_{60} and PDINH/$C_{60}NH_3$. (b) Photocurrent response of PDINH, PDINH/C_{60} and PDINH/$C_{60}NH_3$. (c) Photoluminescence spectra of PDINH, PDINH/C_{60} and PDINH/$C_{60}NH_3$.

The EIS results (**Fig. S37a**) were fitted and calculated by the software ZSimpWin 3.60 as the following equivalent circuit model. In this diagram, R_L is bulk electrolyte resistance, R_d is electrochemical double-layer resistance, R_{sc} is space charge layer resistance, and the smaller R_{sc} induces the facile electrons transmission through the space charge layer. PDINH/$C_{60}NH_3$ exhibits the smallest R_{sc}, suggesting a rapid charge migration (**Table S3**). As shown in **Fig. S37b**, PDINH/$C_{60}NH_3$ films on FTO substrates exhibit water oxidation photocurrents of 13.6 μA cm^{-2} at 1.23 V *vs* RHE under visible light illumination, which is ~6 times higher than that of pure PDINH (2.3 μA cm^{-2} at 1.23 V *vs* RHE). This increased photocurrent indicated that the introduction of $C_{60}NH_3$ can lower the electron transfer barrier, thus promoting the migration of photogenerated charge carriers. Also, compared with the pristine PDINH and PDINH/C_{60}, the photoluminescence (PL) intensity of PDINH/$C_{60}NH_3$ remarkably decreases (**Fig. S37c**), reflecting that charge recombination is significantly inhibited under strong internal electric field.

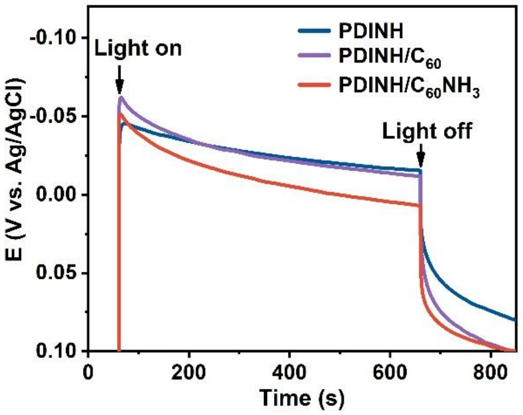

Figure S38. The open-circuit photovoltage measurement. The rise and decay of photoinduced potential of PDINH and PDINH/$C_{60}NH_3$.

The open-circuit photovoltage (OCP) was measured to evaluate the time constants for hole interfacial extraction (τ_h). The test was conducted on a Shanghai Chenhua CHI760E electrochemical workstation with a three-electrode system, and 0.2 M Na_2SO_4 aqueous solution containing 10 vol % methanol was used as the electrolyte. The time for light on lasted for 600 s, then the light was turned off and recording the change in photovoltage [27]. PDINH/$C_{60}NH_3$ shows faster voltage decay than that of the pure PDINH, indicating the more efficient hole interfacial extraction in PDINH/$C_{60}NH_3$ system, and this will facilitate the transfer of more holes to the surface to participate in the water oxidation reaction.

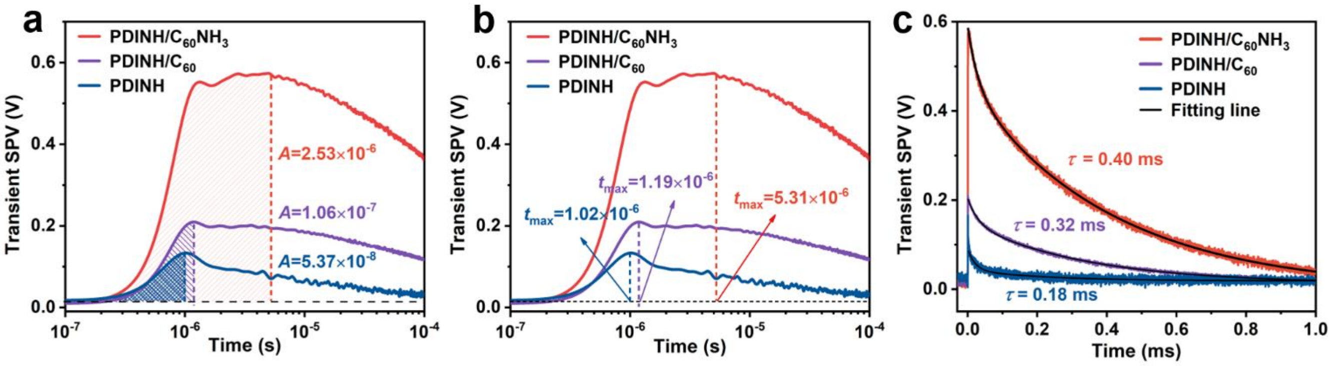

Figure S39. TPV dynamic analysis for PDINH, PDINH/C_{60} and PDINH/$C_{60}NH_3$. (a) Amount of charge extraction (A). (b) The maximum charge extraction time (t_{max}). (c) The attenuation constants (τ) estimated from TPV spectra. τ is the average decay lifetime of the transient photovoltage (**Table S5**).

As shown in **Fig. S39a**, the amount charge extraction (A) of PDINH, PDINH/C_{60} and PDINH/$C_{60}NH_3$ are 5.37×10^{-8}, 1.06×10^{-7} and 2.53×10^{-6}, respectively. **Fig. S39b** indicates the charge extraction time (t_{max}) of PDINH, PDINH/C_{60} and PDINH/$C_{60}NH_3$ are 1.02×10^{-6} s, 1.19×10^{-6} s and 5.31×10^{-6} s respectively. **Fig. S39c** indicates the fitted TPV decay time constant (τ) of 0.18 ms for PDINH, 0.32 ms for PDINH/C_{60} and 0.40 ms for PDINH/$C_{60}NH_3$. As a result, the number of effective

charges (A_e) remaining on the surface (after the charge extraction and recombination) can be calculated by the following formula [28]:

$$A_e = \frac{A \cdot \tau}{t_{max}}$$

Thus, the A_e of PDINH/$C_{60}NH_3$, PDINH/C_{60} and PDINH are determined to be 1.93 $\times 10^{-4}$, 2.85 $\times 10^{-5}$ and 9.73 $\times 10^{-6}$, respectively.

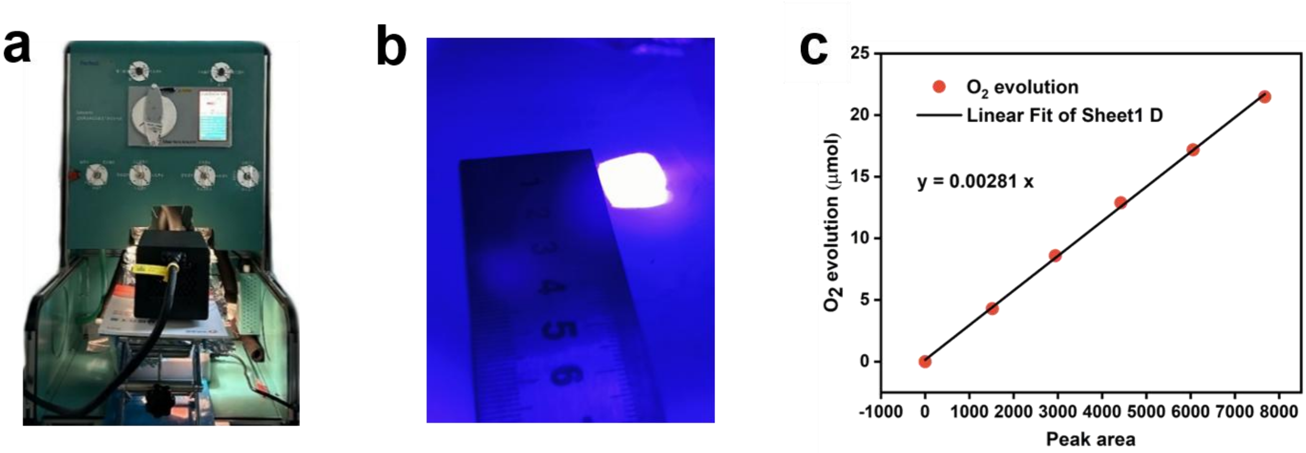

Figure S40. Photocatalytic reaction equipment and O_2 calibration curve. (a) Photograph of the photocatalytic on-line analytical system (Labsolar-6A, Beijing Perfectlight). (b) Xenon lamp irradiation area (1.0 × 1.0 cm^2) during the AQE test. (c) Calibration curve for oxygen production.

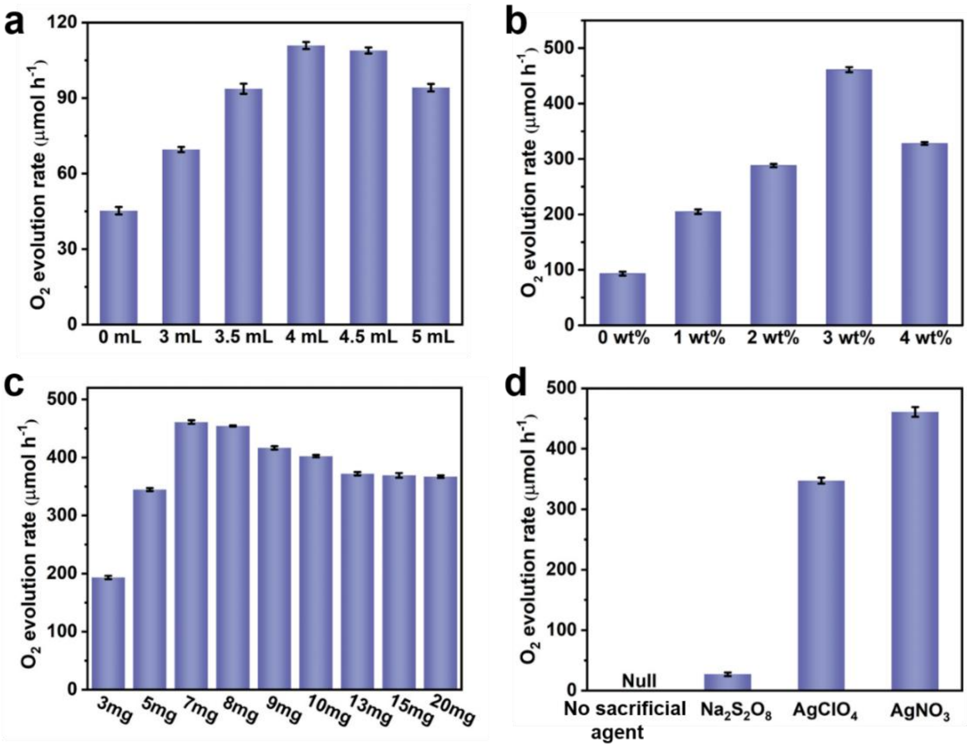

Figure S41. Optimization of oxygen evolution performance for PDINH/$C_{60}NH_3$ under different conditions. (a) Different added amounts of $C_{60}NH_3$ to PDINH. (b) Different deposited amounts of

$Co(OH)_2$. (c) The photocatalytic O_2 evolution rate with different dosages of PDINH/$C_{60}NH_3$. (d) Different electron sacrificial agents.

As shown in **Fig. S41a**, by optimizing the component ratios of $C_{60}NH_3$, the O_2 evolution rate achieved a maximum value when 4 mL of $C_{60}NH_3$ (0.2 mg mL^{-1}) was used to form hydrogen bonding controlled PDINH/$C_{60}NH_3$ assembly. In the presence of $AgNO_3$ as electron sacrifice agent and 3 wt% $Co(OH)_2$ as co-catalyst, the O_2 evolution rate can be optimized to ~450 μmol h^{-1} (**Fig. S41b** and **S41d**). The catalyst dosages during O_2 evolution reaction were also studied (**Fig. S41c**). 7 mg of catalyst was the optimal amount, meaning that such amount of PDINH/$C_{60}NH_3$ reaches saturated light absorption, while the excessive photocatalyst could increase the light scattering and also decrease active sites due to aggregation.

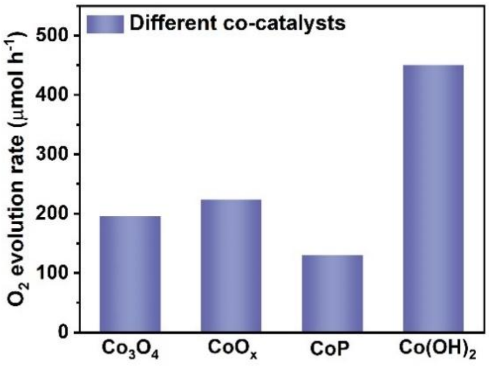

Figure S42. Comparison of oxygen evolution performance using different cobalt-based co-catalysts.

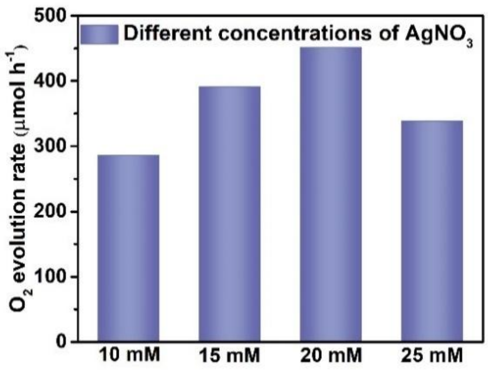

Figure S43. Effect of $AgNO_3$ concentration on oxygen evolution activity.

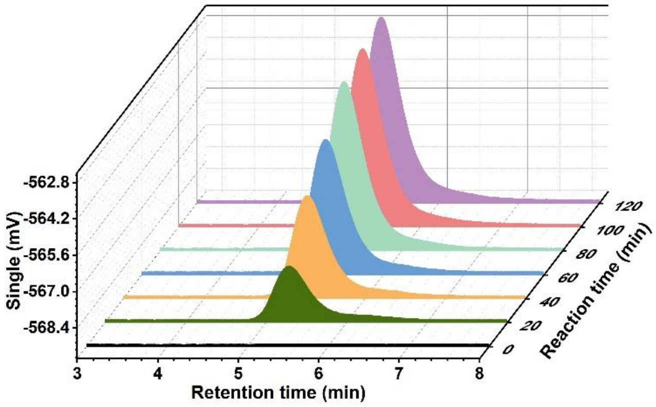

Figure S44. Oxygen evolution detected by gas chromatography. The increasing oxygen area in the gas chromatogram within 2 h of reaction.

The gas chromatogram proves that the peak area of oxygen has been increasing during the course of photocatalytic reaction, while no other peaks were detected.

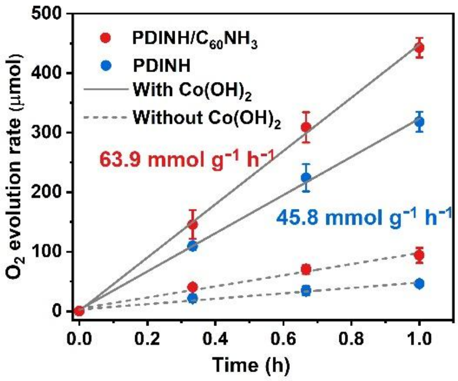

Figure S45. The time-dependent O_2 evolution of PDINH and PDINH/$C_{60}NH_3$. Dashed line denotes no co-catalyst and solid line denotes with co-catalyst.

As shown in **Fig. S45**, in the absence of co-catalyst, the oxygen evolution of PDINH/$C_{60}NH_3$ is 13.3 mmol g^{-1} h^{-1}, which is still ~2 times higher than that of the pristine PDINH (6.4 mmol g^{-1} h^{-1}). With the help of $Co(OH)_2$, the oxygen evolution rate of PDINH/$C_{60}NH_3$ increases to 63.9 mmol g^{-1} h^{-1} and that of PDINH increases to 45.8 mmol g^{-1} h^{-1}, this indicates that the introduction of $C_{60}NH_3$ plays a dominant role in the performance improvement, independent of the co-catalyst.

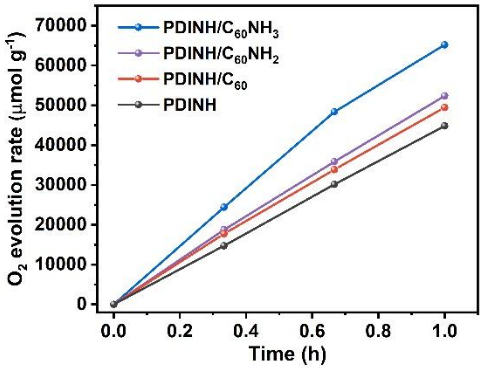

Figure S46. Comparison of oxygen evolution performance of different PDINH-based composites. The time-dependent O_2 evolution of PDINH, PDINH/C_{60}, PDINH/$C_{60}NH_2$ and PDINH/$C_{60}NH_3$.

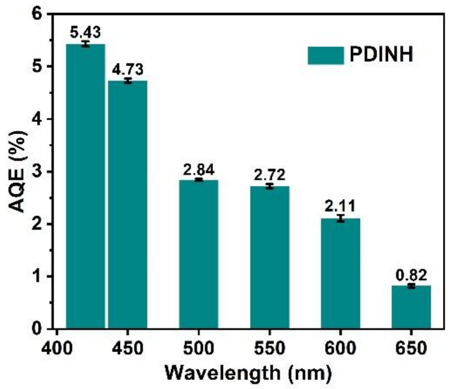

Figure S47. The AQE of photocatalytic O_2 evolution of PDINH. The AQE values of pure PDINH at 420 nm, 450 nm, 500 nm, 550 nm, 600 nm and 650 nm.

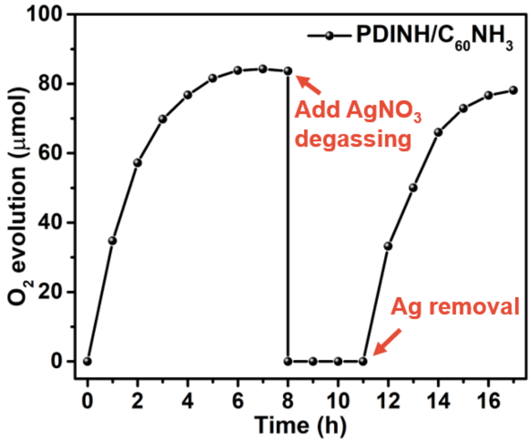

Figure S48. Long-term photocatalytic reaction test. Photocatalytic oxygen evolution of PDINH/$C_{60}NH_3$ for 8 hours.

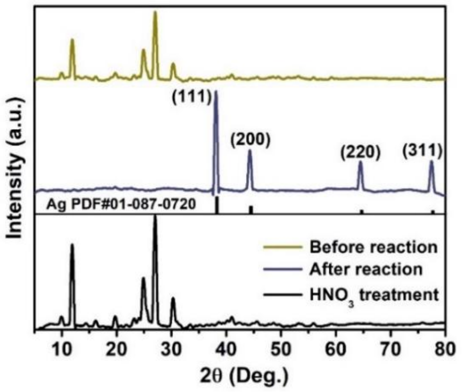

Figure S49. XRD characterization of post-reaction catalyst.

As evidenced by XRD analysis (Fig. S49), after prolonged oxygen evolution reaction, the recycled PDINH/$C_{60}NH_3$ sample displays distinct diffraction peaks corresponding to Ag (JCPDS 01-087-0720), suggesting surface coverage by Ag nanoparticles. After treatment with HNO3 (0.1 mol L^{-1}), the original XRD pattern of PDINH/C60NH3 is completely restored, and the oxygen evolution activity is simultaneously recovered (Fig. S48).

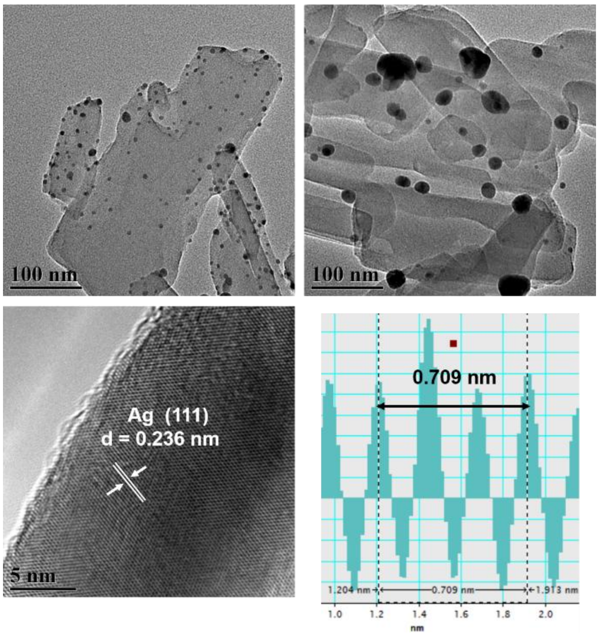

Figure S50. TEM and HRTEM characterizations of post-reaction catalyst.

The TEM and HRTEM results clearly demonstrate the presence of abundant Ag nanoparticles distributed across the catalyst surface, where the dominantly observerd lattice spacing of 0.236 nm is attributed to the Ag (111) plane (Fig. S50). The intense Ag 3d XPS signals reveal extensive surface coverage by Ag nanoparticles, which results in almost undetectable XPS signals of $Co(OH)_2$ co-catalyst (Fig. S51).

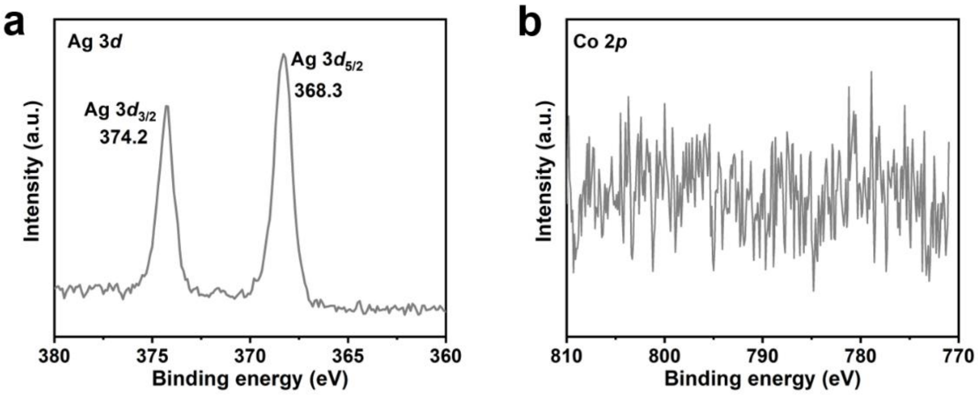

Figure S51. XPS characterization of post-reaction catalyst. Ag 3d and Co 2p spectra of the recycled PDINH/$C_{60}NH_3$ sample.

Supplemental Tables

Table S1. Elemental analysis of PDINH and PDINH/$C_{60}NH_3$ (wt%: weight ratio).

Sample	C (wt%)	N (wt%)	O (wt%)	H (wt%)	N/C	H/C
PDINH	72.835	6.460	17.849	2.560	0.088	0.035
PDINH/$C_{60}NH_3$	69.048	9.308	18.491	2.944	0.134	0.043
$C_{60}NH_3$	51.874	21.360	/	6.591	0.411	0.127

Table S2. XPS analysis results of C/N/O elements for PDINH and PDINH/$C_{60}NH_3$ (at%: atom ratio).

Sample	C (at%)	N (at%)	O (at%)	N/C
PDINH	77.14	4.18	18.68	0.054
PDINH/$C_{60}NH_3$	55.35	5.27	39.38	0.095

Table S3. The TR-PL spectra data for PDINH, PDINH/C_{60} and PDINH/$C_{60}NH_3$ [a].

Sample	τ_1 (ns)	B_1	τ_2 (ns)	B_2	τ_{avg} (ns)
PDINH	0.1763	0.179	1.0866	0.02	0.5475
PDINH/C_{60}	0.1570	0.199	1.0512	0.017	0.4823
PDINH/$C_{60}NH_3$	0.1320	0.288	0.9327	0.015	0.3860

[a] All the samples were prepared on quartz. The τ_{avg} was calculated using the equation: $\tau_{avg} = \Sigma B_i \tau_i^2 / (\Sigma B_i \tau_i)$.

Table S4. The fitted parameters of electrochemical impedance spectra.

Sample	R_L (Ω)	R_d (Ω)	R_{sc} (Ω)
PDINH	15.03	3674	66680

$PDINH/C_{60}$	16.38	5283	40530
$PDINH/C_{60}NH_3$	10.94	149.9	3811

Table S5. Two-exponential decay time constant of transient photovoltage.

Sample	τ_1 (ms)	τ_2 (ms)	A_1	A_2	τ (ms)
PDINH	0.01733	0.20817	0.61005	0.30006	0.18
$PDINH/C_{60}$	0.03007	0.34004	0.05444	0.13659	0.32
$PDINH/C_{60}NH_3$	0.03148	0.41225	0.23551	0.81452	0.40

The average decay time τ was calculated by the following formula:

$$\tau = \frac{A_1 \times \tau_1^2 + A_2 \times \tau_2^2}{A_1 \times \tau_1 + A_2 \times \tau_2}$$

Table S6. The AQE of $PDINH/C_{60}NH_3$.

Wavelength (nm)	420	450	500	550	600	650	700
O_2 evolution (μmol)	2.86	2.01	2.05	2.53	3.98	2.66	1.16
Light intensity (mW/cm^2)	7.65	6.52	9.13	14.65	11.80	13.33	12.48
Irradiation time (h)	1	1	1	1	1	1	1
Irradiation area (cm^2)	1	1	1	1	1	1	1
AQE (%)	11.83	9.12	5.97	4.17	7.49	4.08	1.77

The calculation of AQE, taking λ = 420 nm for example:

$$\mathrm{N} = \frac{\mathrm{E}\lambda}{\mathrm{hc}} = \frac{7.65 \times 1.0 \times 1.0 \times 10^{-3} \times 1 \times 3600 \times 420 \times 10^{-9}}{6.626 \times 10^{-34} \times 3 \times 10^{8}}$$
$$= 5.82 \times 10^{19}$$

$$\mathrm{AQE} = \frac{4 \times \text{the number of evolved } \mathrm{O_2} \text{ molecule}}{\mathrm{N}} \times 100\%$$
$$= \frac{4 \times 6.02 \times 10^{23} \times 2.86 \times 10^{-6}}{5.82 \times 10^{19}} \times 100\%$$
$$= 11.83\%$$

Where N is the incident photon quantity; E is the incident energy; λ is the wavelength of light; h is Planck's constant (6.626×10^{-34}); and c is the speed of light (3×10^{8} m/s).

Table S7. Summary and comparison of photocatalytic oxygen-producing materials.

Photocatalysts	Rate (mmol g^{-1} h^{-1})	Light Source	Catalyst dosage (mg)	Co-catalyst	Sacrificial agent	AQE at 420 nm (%)	Ref.
PDINH/$C_{60}NH_3$	63.9	≥420 nm	7	$Co(OH)_2$	$AgNO_3$	11.83<br>4.08 (650nm)	This work
IrOx-am@TiO_2	0.1436	AM 1.5	100	None	$AgNO_3$	7.82 (LED-425)	[29]
BpCo-COF-1	0.152	≥420 nm	10	Co	$AgNO_3$	0.46	[30]
r-CTF NSs	0.2474	≥420 nm	50	$Co(NO_3)_2$	$AgNO_3$	5.6	[31]
4Clper-POPs	0.71417	>420 nm	50	$Co(NO_3)_2$	$AgNO_3$	4.13 (450 nm)	[32]
Co_4O_4/pGO/$PbCrO_4$	~1	≥420 nm	100	None	$Fe(NO_3)_3$	~10.5 (500 nm)	[33]
CTF-0-I	0.226	Full arc	100	None	$AgNO_3$	5.2	[34]
PDIP	3.224	≥420 nm	25	None	$AgNO_3$	3.86 (450 nm)	[35]
Oxamide-PDI	5.110	Full spectrum	25	None	$AgNO_3$	2.15	[36]
Co-PDI	5.53	≥420 nm	20	None	$AgNO_3$	7.48	[37]
PDI/Co_3O_4/Pt	24.4	≥420 nm	15	None	$AgNO_3$	6.9	[38]
PDI-NH	40.6	≥420 nm	15	$Co(OH)_2$	$AgNO_3$	10.4 (400 nm)	[1]
$BiVO_4$ NFs	~1.31	≥420 nm	100	None	$AgNO_3$	41.2	[39]
Ni-TAPP-COF-BF_2	1.404	AM 1.5G	5	None	$AgNO_3$	1.27 (450 nm)	[40]
TAPT-Bpy-COF	0.483	≥420 nm	5	Co	$AgNO_3$	7.6	[41]
g-$C_{54}N_6$-COF	0.051	≥420 nm	50	Co	$AgNO_3$	/	[42]
CoTPP-$CoBpy_3$	7.3	300 W Xe<br>AM 1.5G	5	None	$AgNO_3$	4.65 (450 nm)	[43]

Supplemental References

[1] Sheng Y, Li W, Xu L *et al*. High photocatalytic oxygen evolution via strong built-in electric field induced by high crystallinity of perylene imide supramolecule. *Adv Mater* 2022; **34**: 2102354.

[2] Li J, Zhao F, Wang T *et al*. Ethylenediamine functionalized fullerene nanoparticles as independent electron transport layers for high-efficiency inverted polymer solar cells. *J Mater Chem A* 2017; **5**: 947-51.

[3] Chi Z, Chen H, Zhao Q *et al*. Ultrafast carrier and phonon dynamics in few-layer 2H–$MoTe_2$. *J Chem Phys* 2019; **151**: 114704.

[4] Zhang G, Zang S, Wang X *et al*. Layered $Co(OH)_2$ deposited polymeric carbon nitrides for photocatalytic water oxidation. *ACS Catal* 2015; **5**: 941-7.

[5] Kresse G and Hafner J. Ab initio molecular dynamics for open-shell transition metals. *Phys Rev B* 1993; **48**: 13115-8.

[6] Kresse G and Furthmüller J. Efficient iterative schemes for ab initio total-energy calculations using a plane-wave basis set. *Phys Rev B* 1996; **54**: 11169-86.

[7] Kresse G and Furthmüller J. Efficiency of ab-initio total energy calculations for metals and semiconductors using a plane-wave basis set. *Comp. Mater. Sci* 1996; **6:** 15-50.

[8] Blöchl P.E. Projector augmented-wave method. *Phys Rev B* 1994; **50**: 17953-79.

[9] Grimme S. Accurate description of van der Waals complexes by density functional theory including empirical corrections. *J Comput Chem* 2004; **25**: 1463-73.

[10] Lu T and Chen F. Multiwfn: A multifunctional wavefunction analyzer. *J Comput Chem* 2012; **33**: 580-92.

[11] Humphrey W. Dalke A and Schulten K. VMD: Visual molecular dynamics. *J. Mol. Graph* 1996; **14**: 33-38.

[12] Hunter C.A and Sanders J.K.M. The nature of .pi.-.pi. interactions. *J Am Chem Soc* 1990; **112**: 5525-34.

[13] Liu D, Wang J, Bai X *et al*. Self-assembled PDINH supramolecular system for photocatalysis under visible light. *Adv Mater* 2016; **28**: 7284-90.

[14] Jing J, Li J, Su Y *et al*. Non-covalently linked donor-acceptor interaction enhancing photocatalytic hydrogen evolution from porphyrin assembly. *Appl Catal B: Environ* 2023; **324**: 122284.

[15] Grande V, Soberats B, Herbst S *et al*. Hydrogen-bonded perylene bisimide J-aggregate aqua material. *Chem Sci* 2018; **9**: 6904-11.

[16] Ai L, Xiang W, Li ZW *et al*. Hydrogen bond-induced flexible and twisted self-assembly of functionalized carbon dots with customized-color circularly polarized luminescence. *Angew Chem Int Ed* 2024; **63**: e202410988.

[17] Jiang Y, Wang C, Dong T *et al*. Fullerene C_{70}-encapsulated tetrathiafulvalene-Co porphyrin covalent organic framework: driving multistep charge transfer to boost CO_2 photoreduction. *Adv Sci* 2025; **12**: 2505161.

[18] Guo Y, Ma Z, Niu X *et al*. Bridge-mediated charge separation in isomeric N-annulated perylene diimide dimers. *J Am Chem Soc* 2019; **141**: 12789-96.

[19] Lin C, Kim T, Schultz JD *et al*. Accelerating symmetry-breaking charge separation in a perylenediimide trimer through a vibronically coherent dimer intermediate. *Nat Chem* 2022; **14**: 786-93.

[20] Guldi DM and Prato M. Excited-state properties of C_{60} fullerene derivatives. *Acc Chem Res* 2000; **33**: 695-703.

[21] L. Liu L, Meng H, Chai Y *et al*. Enhancing built-in electric fields for efficient photocatalytic hydrogen evolution by encapsulating C_{60} fullerene into zirconium-based metal-organic frameworks. *Angew Chem Int Ed* 2023; **62**: e202217897.

[22] Shi Y, Li J, Mao C *et al*. Van Der Waals gap-rich BiOCl atomic layers realizing efficient, pure-water CO_2-to-CO photocatalysis. *Nat Commun* 2021; **12**: 5923

[23] Shu C, Yang X, Liu L *et al.* Mixed-linker strategy for the construction of sulfone-containing D-A-A covalent organic frameworks for efficient photocatalytic hydrogen peroxide production. *Angew Chem Int Ed* 2024; **63**: e202403926.

[24] Jiang W, Ni C, Zhang L *et al*. Tuning the anisotropic facet of lead chromate photocatalysts to promote spatial charge separation. *Angew Chem Int Ed* 2022; **61**: e202207161.

[25] Li Z, Zhang L, Liu Y *et al*. Surface-polarity-induced spatial charge separation boosts photocatalytic overall water splitting on GaN nanorod arrays. *Angew Chem Int Ed* 2020; **59**: 935-42.

[26] Zhao D, Wang Y, Dong C.-L *et al.* Boron-doped nitrogen-deficient carbon nitride-based Z-scheme heterostructures for photocatalytic overall water splitting. *Nat Energy* 2021; **6**: 388-97.

[27] Ge T and Chen J. Evaluating a second time scale for hole extraction in an actual photocatalytic reaction: the method. *J Phys Chem Lett* 2023; **14**: 7477-82.

[28] Chen Q, Mao B, Liu Y *et al*. Designing 2D carbon dot nanoreactors for alcohol oxidation coupled with hydrogen evolution. *Nat Commun* 2024; **15**: 8052.

[29] Yan J, Liu J, Ji Y *et al.* Surface engineering to reduce the interfacial resistance for enhanced photocatalytic water oxidation. *ACS Catal* 2020; **10**: 8742-50.

[30] Chen J, Tao X, Li C *et al*. Synthesis of bipyridine-based covalent organic frameworks for visible-light-driven photocatalytic water oxidation. *Appl Catal B: Environ* 2020; **262**: 118271.

[31] Wang C, Zhang H, Luo W *et al*. Ultrathin crystalline covalent-triazine-framework nanosheets with electron donor groups for synergistically enhanced photocatalytic water splitting. *Angew Chem Int Ed* 2021; **60**: 25381-90.

[32] Li H, Luo X, Chen M *et al.* Porous organic polymers involving chloro-substituted peryleneimide for photocatalytic water oxidation under visible light irradiation. *Chem Eng J* 2022; **443**: 136463.

[33] Jiang W, Zhang L, Ni C *et al*. Graphene mediates charge transfer between lead chromate and a cobalt cubane cocatalyst for photocatalytic water oxidation. *Angew Chem Int Ed* 2023; **62**: e202302575.

[34] Kong D, Han X, Xie J *et al*. Tunable covalent triazine-based frameworks (CTF-0) for visible-light-driven hydrogen and oxygen generation from water splitting. *ACS Catal* 2019; **9**: 7697-707.

[35] Zhang Z, Chen X, Zhang H *et al*. A highly crystalline perylene imide polymer with the robust built-in electric field for efficient photocatalytic water oxidation. *Adv Mater* 2020; **32**: 1907746.

[36] Liu D, Yang X, Chen P *et al*. Rational design of PDI-based linear conjugated polymers for highly effective and long-term photocatalytic oxygen evolution. *Adv Mater* 2023; **35**: 2300655.

[37] Lin Z, Wang Y, Peng Z *et al*. Single-metal atoms and ultra-small clusters manipulating charge carrier migration in polymeric perylene diimide for efficient photocatalytic oxygen production. *Adv Energy Mater* 2022; **12**: 2200716.

[38] Li W, Wei Z, Sheng Y *et al*. Dual cocatalysts synergistically promote perylene diimide polymer charge transfer for enhanced photocatalytic water oxidation. *ACS Energy Lett* 2023; **8**: 2652-60.

[39] Philo D, Luo S, He C *et al*. Lattice distortion engineering over ultrathin monoclinic $BiVO_4$ nanoflakes triggering AQE up to 69.4% in visible-light-driven water oxidation. *Adv Funct Mater* 2022; **32**: 2206811.

[40] Borse R.A, Tan Y.-X, Lin J *et al*. Coupling electron transfer and redox site in boranil covalent organic framework toward boosting photocatalytic water oxidation. *Angew Chem Int Ed* 2024; **63**: e202318136.

[41] Chen H, Gardner A.M, Lin G *et al*. Triazine-based covalent organic framework for photocatalytic water oxidation: the role of bipyridine ligand and cobalt coordination. *J Phys Chem C* 2023; **127**: 14137-45.

[42] Xu J, Yang C, Bi S *et al*. Vinylene-linked covalent organic frameworks (COFs) with symmetry-tuned polarity and photocatalytic activity. *Angew Chem Int Ed* 2020; **59**: 23845-53.

[43] Zhou E, Zhang X, Zhu L *et al*. Ultrathin covalent organic framework nanosheets for enhanced photocatalytic water oxidation. *Sci Adv* 2024; **10**: eadk8564.
